# Establishment and application of an iELISA detection method for measuring apical membrane antigen 1 (AMA1) antibodies of *Toxoplasma gondii* in cats

**DOI:** 10.1186/s12917-023-03775-1

**Published:** 2023-11-03

**Authors:** Yafan Gao, Yu Shen, Jiyuan Fan, Haojie Ding, Bin Zheng, Haijie Yu, Siyang Huang, Qingming Kong, Hangjun Lv, Xunhui Zhuo, Shaohong Lu

**Affiliations:** 1 School of Basic Medical Sciences and Forensic Medicine, Hangzhou Medical College, Hangzhou, 310013 China; 2 Engineering Research Center of Novel Vaccine of Zhejiang Province, Hangzhou Medical College, Hangzhou, China; 3 Key Laboratory of Bio-Tech Vaccine of Zhejiang Province, Hangzhou Medical College, Hangzhou, China; 4 Jiaxing Vocational & Technical College, Jiaxing, 314036 China; 5 Institute of Comparative Medicine, College of Veterinary Medicine, Yangzhou University, Yangzhou, 225009 China

**Keywords:** *Toxoplasma gondii*, Apical membrane antigen 1, Cats, Detection, ELISA

## Abstract

**Background:**

Diseases caused by *Toxoplasma gondii* (*T. gondii*) have introduced serious threats to public health*.* There is an urgent need to develop a rapid detection method for *T. gondii* infection in cats, which are definitive hosts. Recombinant apical membrane antigen 1 (rAMA1) was produced in a prokaryotic expression system and used as the detection antigen. The aim of this study was to evaluate and optimize a reliable indirect enzyme-linked immunosorbent assay (iELISA) method based on rAMA1 for the detection of antibodies against *T. gondii* in cats.

**Results:**

The rAMA1-iELISA method was developed and optimized by the chessboard titration method. There were no cross-reactions between *T. gondii*-positive cat serum and positive serum for other pathogens, indicating that rAMA1-iELISA could only detect *T. gondii* in most cases. The lowest detection limit of rAMA1-iELISA was 1:3200 (dilution of positive serum), and the CV of repeated tests within batches and between batches were confirmed to be less than 10%. The results of 247 cat serum samples detected by rAMA1-iELISA (kappa value = 0.622, *p* < 0.001) were in substantial agreement with commercial ELISA. The ROC curve analysis revealed the higher overall check accuracy of rAMA1-iELISA (sensitivity = 91.7%, specificity = 93.6%, AUC = 0.956, 95% CI 0.905 to 1.000) than GRA7-based iELISA (sensitivity = 91.7%, specificity = 85.5%, AUC = 0.936, 95% CI 0.892 to 0.980). Moreover, the positive rate of rAMA1-iELISA (6.5%, 16/247) was higher than that of GRA7-based iELISA (3.6%, 9/247) and that of commercial ELISA kit (4.9%, 12/247).

**Conclusion:**

The iELISA method with good specificity, sensitivity, and reproducibility was established and can be used for large-scale detection of *T. gondii* infection in clinical cat samples.

**Supplementary Information:**

The online version contains supplementary material available at 10.1186/s12917-023-03775-1.

## Background

*Toxoplasma gondii* (*T. gondii*), an obligate intracellular protozoan parasite that is responsible for toxoplasmosis, is widely epidemic in almost all warm-blooded animals, including humans [1]. More than one-third of the world’s population is seropositive for *T. gondii* [2]. The high frequency of *T. gondii* infection has become a public concern and threat to the global population, especially immunocompromised patients and pregnant women [3, 4]. Cysts and oocysts are the principal propagation sources for toxoplasmosis [5]. In addition, felines, such as cats, are the definitive hosts where *T. gondii* undergoes sexual reproduction leading to oocyst excretion in the feces [6]. Given the increasing population of pet cats [7], which are in close contact with humans, and the fact that cats play a major role in the transmission of *T. gondii*, pet cats may be an important source of human toxoplasmosis [8]. There is an incalculable number of stray cats in the environment [9], increasing public health problems and disease exchange between stray and pet cat populations. Therefore, the development of simple, inexpensive, and sensitive diagnostic tests for *T. gondii* detection in cats is crucial to prevent and control toxoplasmosis in humans and cats.

For the diagnosis of *T. gondii*, the main applied methods are pathogen inspections, molecular biology tests and immunological assays [10]. Pathogenic diagnostics is a complex and time-consuming method that includes direct microscopy, trophozoite examination, and animal infection. It is accurate but not suitable for clinical diagnosis in community hospitals and clinics. Molecular biology tests, such as nucleic acid molecular hybridization, polymerase chain reaction (PCR), isothermal amplification technology, and gene sequencing technology, are widely used with preferable specificities and sensitivities, but instruments and technicians are vital for these applications [11]. Immunological assays, especially serologic tests such as the Sabin-Feldman dye test (DT), enzyme-linked immunosorbent assay (ELISA), immunosorbent agglutination assay (ISAGA), indirect hemagglutination assay (IHA) and Western blot (WB), are often used for the detection of antibodies against *T. gondii* in humans and animals [12]. Among them, ELISA is most suitable for clinical use and can process a large number of samples in a short period of time with good specificity and sensitivity.

*T. gondii* apical membrane antigen 1 (AMA1), as the microneme protein, is involved in the initial stages of parasite invasion, including recognition, attachment, and subsequent entry into the host cell [13–15], and promotes tachyzoite replication [16]. AMA1 is expressed and secreted in almost every life stage of the parasite and shows strong immunogenicity and immune protection against acute and chronic toxoplasmosis in mice [14, 17, 18]. Furthermore, studies have shown that AMA1 strongly interacts with specific anti-*T. gondii* IgG (99.4%) and IgM (80.0%) antibodies, and the ELISA method constructed by recombinant AMA1 protein could be applied to mouse and human sera [19], while whether this protein would also be an ideal candidate for the development of a diagnostic method for cat toxoplasmosis remains unknown.

In this study, a reliable indirect enzyme-linked immunosorbent assay (iELISA) method based on rAMA1 was evaluated and optimized for the detection of antibodies against *T. gondii* in cats. In addition, we investigated the infection of stray cats and domestic cats in Jiaxing by using the established iELISA method.

## Results

### Expression and purification of rAMA1 proteins

**Fig. 1:**
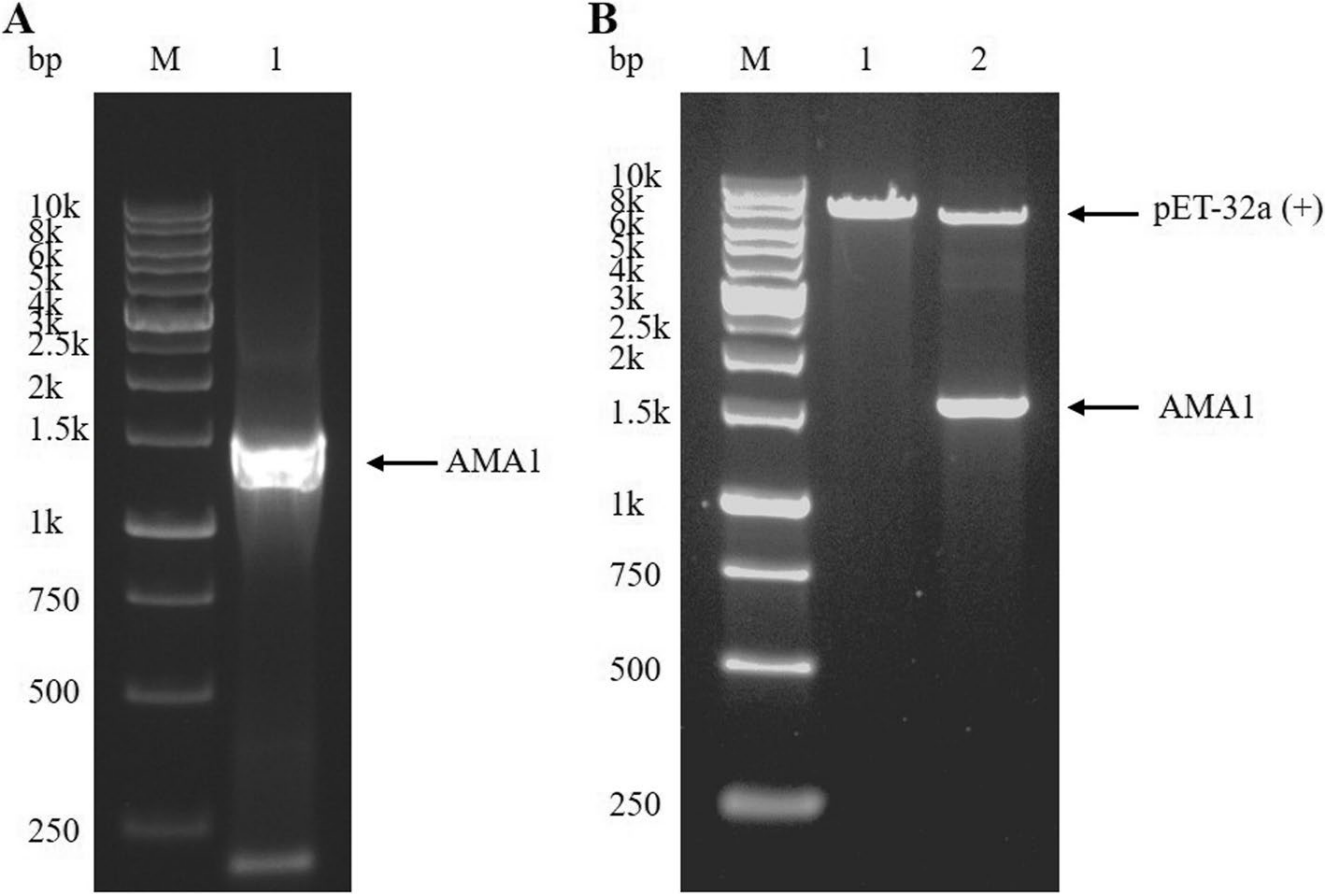
Identification of PCR amplification products and enzyme digestion products. **A** PCR amplification of AMA1 with the primers AMA1-F/AMA1-R. M: 250–10000 bp DNA Marker; Lane 1: Apical membrane antigen 1 (AMA1) amplicon length 1512 bp (arrow); **B** Enzyme digestion identification of recombinant plasmid pET-32a ( +)-AMA1. M: 250–10000 bp DNA Marker; Lane 1: The empty plasmid pET-32a ( +) was used as a blank control; Lane 2: Two distinct bands were obtained by enzyme digestion of pET-32a ( +)-AMA1: Expression vector pET-32a ( +) length 5866 bp (arrow) and AMA1 strip length 1512 bp (arrow)

**Fig. 2:**
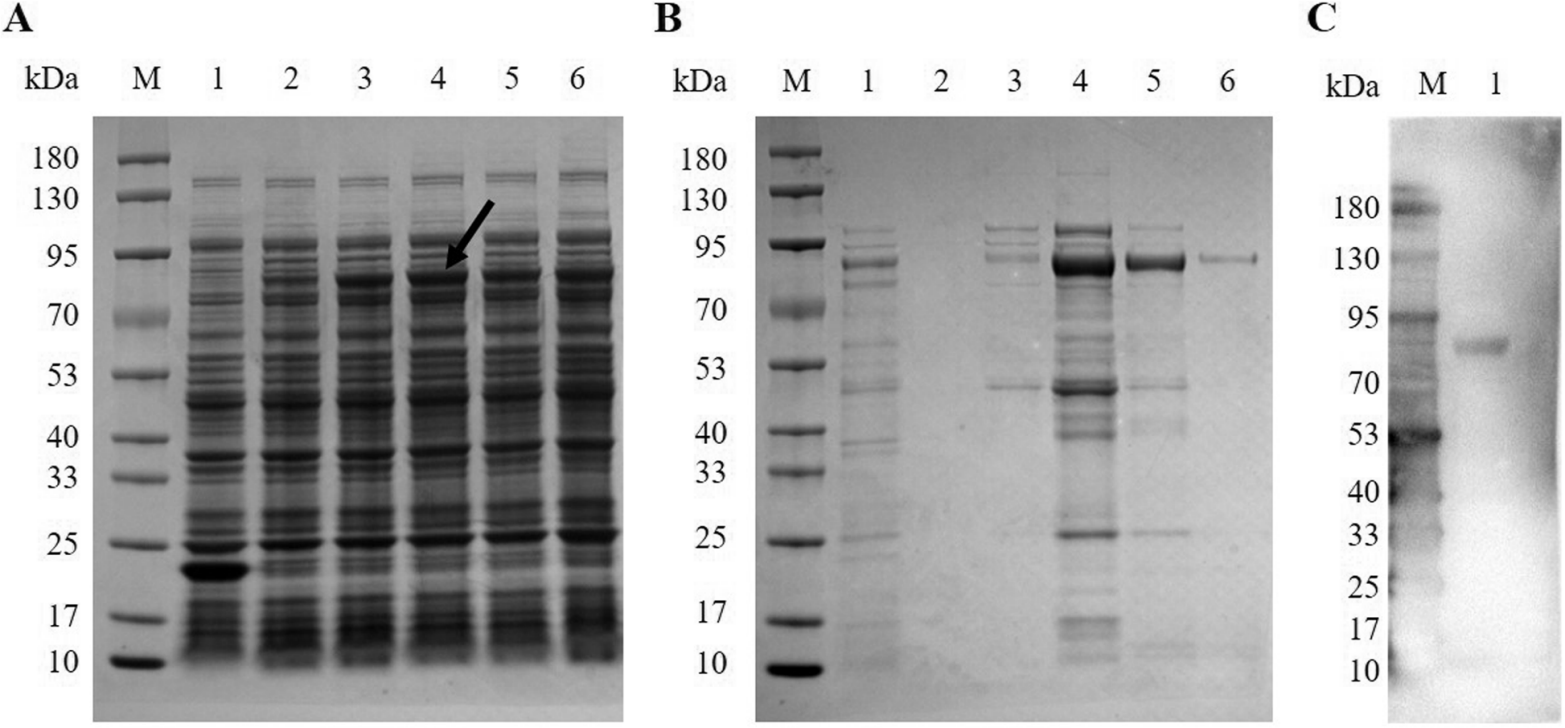
Expression and analysis of recombinant Apical membrane antigen 1 (rAMA1) of *Toxoplasma gondii*. **A** Sodium dodecyl sulfate-poly-acrylamide gel electrophoresis (SDS-PAGE) analysis of rAMA1 incubated at different times. M: Marker; Lane 1: Induced control culture of cells with empty vector pET-32a ( +); lane 2–6: Expression of rAMA1 after 0, 2, 4, and 6 h of induction separately at 37 ℃. The arrowhead indicates that the highest expression level of rAMA1 was 4 h after induction at 37 ℃. **B** SDS-PAGE analysis of rAMA1 eluted with imidazole at different concentrations. M: Marker; Lane 1: Control culture of unpurified cell lysate after ultrasound; lane 2–6 Elution of rAMA1 with imidazole at 50, 100, 200, 400, 500 mM. **C** Western blot (WB) analysis of rAMA1 with His-tag. M: Marker; lane 1: Mixed multiple 400 mM imidazole-eluted rAMA1

A clear band of nearly 1500 bp was obtained by AMA1 PCR amplification (Fig. 1A). Two clear bands of 5866 bp and 1512 bp were obtained by double enzyme digestion of the *EcoR* I/*Xho* I site of pET-32a ( +)-AMA1 (Fig. 1B), indicating that the recombinant plasmid was successfully constructed. Then, pET-32a ( +)-AMA1 was transformed into competent *E. coli* BL21 (DE3) (CC96107-02, Tolo Biotech, China) cells, and the approximately 74.1 kDa rAMA1 protein was successfully induced by IPTG (I274316, Aladdin, China) with the highest expression level at 4 h for 37 ℃ (Fig. 2A). As shown in Fig. 2B, rAMA1 was purified by using a Ni‐NTA Sefinose ™ Resin column (C600791-0010, Sangon Biotech, China) dodecyl, and the optimal elution concentration of imidazole was 400 mM. Immunoreactivity of rAMA1 was confirmed by western blot (Fig. 2C).

### Optimization of AMA1‑iELISA

**Fig. 3:**
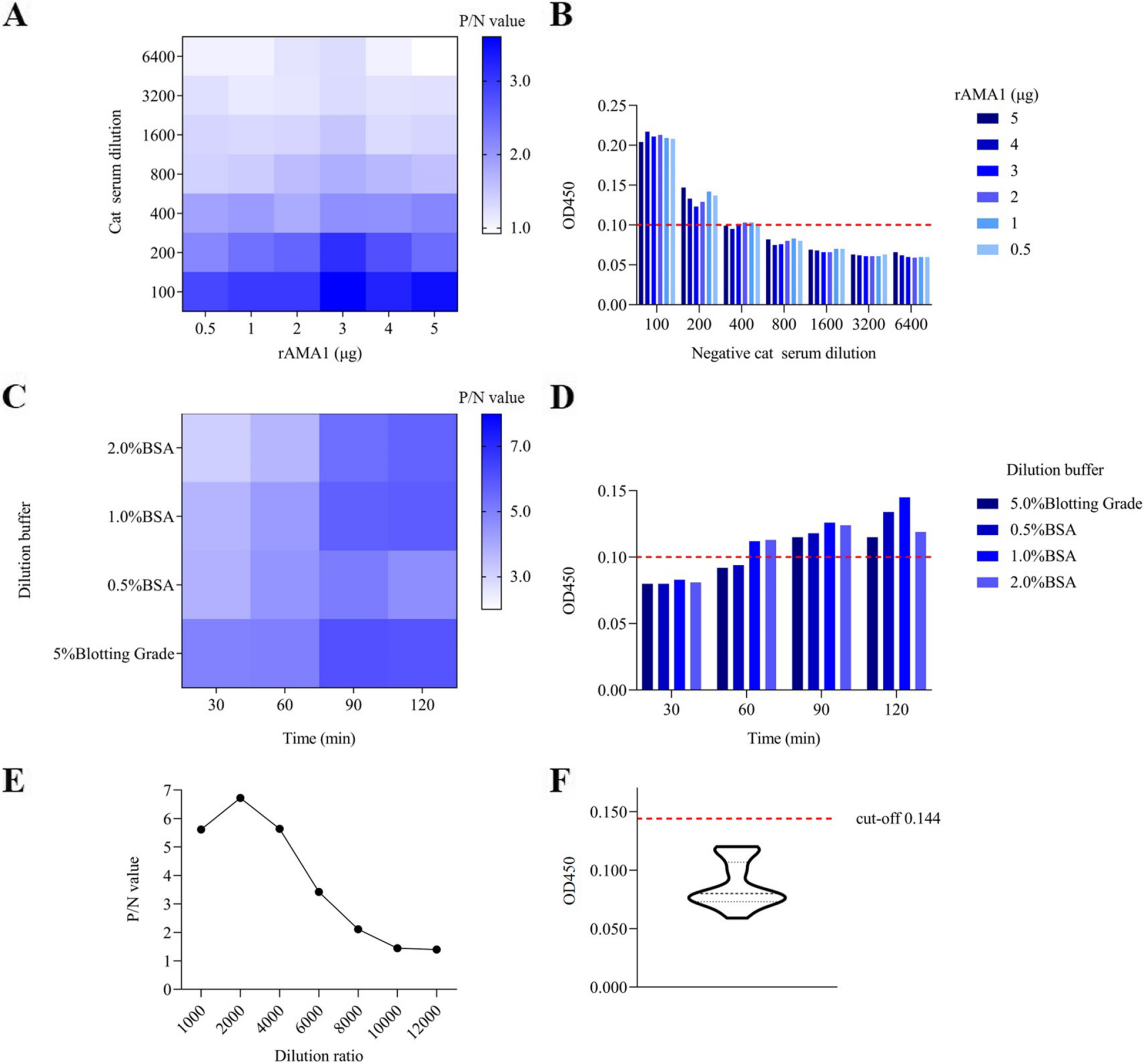
Optimization of rAMA1-iELISA. **A** Optimal dilution ratio of rAMA1 and cat serum. **B** Measuring the optical density value at 450 nm (OD450) of the dilution ratio of rAMA1 and negative cat serum. **C** Optimization of rabbit anti-cat IgG H&L/HRP dilution buffer and incubation time. **D** Measuring the OD450 of negative cat serum with different dilution buffers and incubation times. **E** Optimization of rabbit anti-cat IgG H&L/HRP dilution. **F** Cut-off value of rAMA1-iELISA (cut-off = $\overline{x }$  + 3SD)

The chessboard titration showed that the optimal concentration of rAMA1 was 5 µg per well, and the dilution of cat serum was 1:400 when the value of negative cat serum was less than 0.1 (Fig. 3A and B). Rabbit anti-cat IgG H&L/HRP (K0082R, Solarbio, China) was diluted 1:2000 with 5.0% blotting grade dissolved in PBST (10 mM phosphate buffered saline with 0.05% Tween 20) and incubated for 60 min (Fig. 3C-E). The optimal blocking buffer was 1.0% bovine serum albumin (BSA, ST025, Beyotime Biotechnology, China) dissolved in PBST, and the cat serum dilution buffer was 5.0% Blotting Grade dissolved in PBST and incubated for 60 min. Finally, the TMB substrate solution (P0209, Beyotime Biotechnology, China) was incubated for 10 min (Additional file 1). The cut-off value of rAMA1-iELISA was determined to be 0.144 ($\overline{x }$ = 0.087, SD = 0.019) by calculating the OD450 of 45 negative cat serum of *T. gondii* under optimal conditions (Fig. 3F).

### Cross-reaction, detection limit and intra-and inter-assay precision of rAMA1-iELISA

**Fig. 4:**
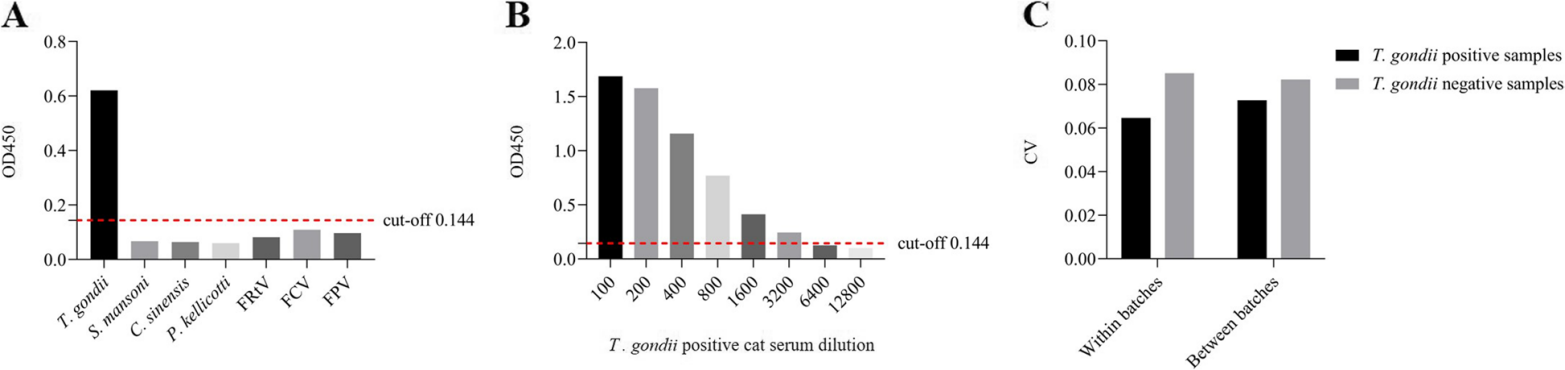
Cross-reaction, detection limit and coefficient of variation (CV) of rAMA1-iELISA. **A** The cross-reactions of rAMA1-iELISA were assessed by detecting six common cat parasites or viruses. **B** The lowest detection limit of rAMA1-iELISA was determined by serial dilutions of cat *T. gondii*-positive serum. **C** CV of repeated tests within batches and between batches

Under the optimized conditions described above, only *T. gondii*-positive serum could be detected by rAMA1-iELISA, indicating that there are no cross-reactions between *T. gondii*-positive cat serum and positive cat serum for other pathogens, including *Spirometra mansoni* (*S. mansoni*), *Clonorchis sinensis* (*C. sinensis*), *Paragonimus kellicotti* (*P. kellicotti*), rhinotracheitis virus (FRtV), calicivirus (FCV) and panleukopenia virus (FPV) (Fig. 4A). As shown in Fig. 4B, the lowest detection limit of rAMA1-iELISA was *T. gondii*-positive cat serum with the dilution rate of 1:3200. Regarding the intra- and inter-assay precision, the coefficient of variation (CVs) of repeated tests within batches and between batches were confirmed to be less than 10% (Fig. 4C), suggesting the good stability of rAMA1-iELISA.

### Clinical application of rAMA1-iELISA

**Fig. 5:**
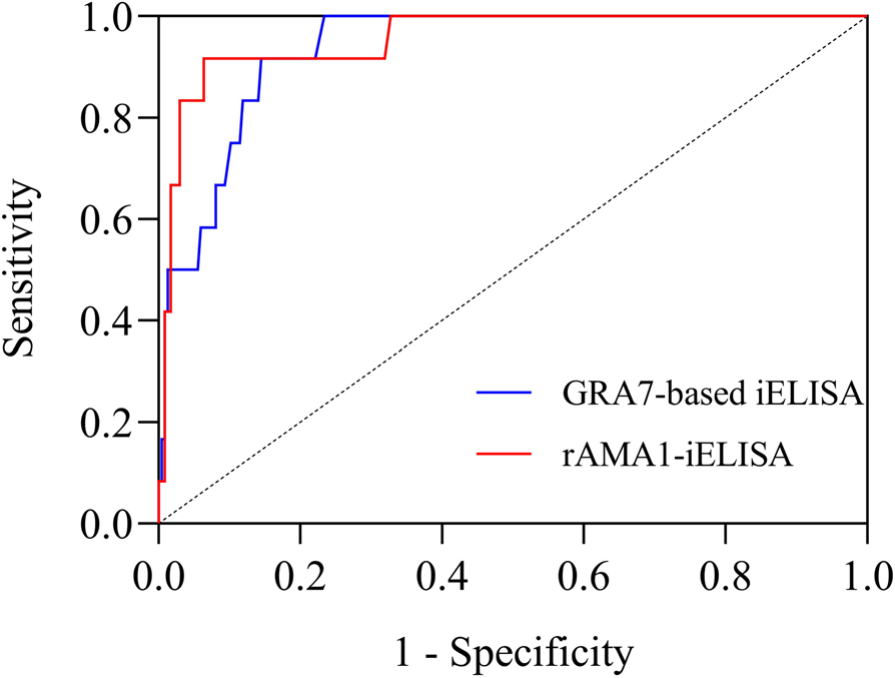
Receiver operating characteristic (ROC) curve analysis of rAMA1-iELISA and GRA7-based iELISA

A total of 247 serum samples, including 11 stray cat samples collected from Jiaxing, were examined by the established rAMA1-iELISA method. The results showed that 13 pet and 3 stray cats were *T. gondii*-positive. The 16 positive and partial negative results obtained by rAMA1-iELISA were further confirmed by WB assay (the results were shown in Additional file 2). The optimal cut-off value was determined to be 0.112 by which sensitivity and specificity of rAMA1-iELISA were 91.7% and 93.6%, respectively, and the receiver operating characteristic (ROC) curve analysis revealed area under the curve (AUC) of 0.956 (95% CI 0.905 to 1.000). The optimal cut-off value of 0.084 that sensitivity and specificity of GRA7-based iELISA were 91.7% and 85.5%, respectively, the ROC curve analysis revealed the AUC of 0.936 (95% CI 0.892 to 0.980) (Fig. 5).

As shown in Table 1, 16/247 and 231/247 cats showed a positive and a negative result, respectively, by rAMA1- iELISA with cut-off value of 0.144. As for GRA7-based iELISA and commercial ELISA kit, the positive and negative results were determined to be 9/247, 238/247 and 12/247, 235/247, respectively. The positive rate of rAMA1-iELISA (6.5%, 16/247) was higher than that of GRA7-based iELISA (3.6%, 9/247) and that of commercial ELISA kit (4.9%, 12/247). Compared with commercial ELISA kit, 9 true positives and 7 false positives were detected in rAMA1-iELISA, while 6 true positives and 3 false positives were found in GRA7-based iELISA. In addition, rAMA1-iELISA was the substantial degree of agreement (kappa value [*κ*] = 0.622, *p* < 0.001) with commercial ELISA kit, GRA7-based iELISA was the moderate degree of agreement (*κ* = 0.553, *p* < 0.001) with commercial ELISA kit.

**Table 1:** Detection of *Toxoplasma gondii* infection in cats by rAMA1-iELISA and GRA7-based iELISA in comparison with commercial ELISA kit

Detection method	rAMA1-iELISA		Total	GRA7-based iELISA		Total
	Positive	Negative		Positive	Negative	
Commercial ELISA kit						
Positive	9	3	12	6	6	12
Negative	7	228	235	3	232	235
Total	16	231	247	9	238	247

## Discussion

With the improvement of living standards, an increasing number of families raise pet cats [20]. Cats are easily infected with *T. gondii*, which excretes infectious oocysts from feces [21], polluting soil and water sources. Thus, it is important for human health to detect whether cats become infected with *T. gondii*. Several common serologic tests, such as DT, ELISA, ISAGA, IHA and WB, were applied for the detection of *T. gondii* infection in cats, and ELISA was one of the most commonly used [22]. Therefore, in this study, an iELISA detection method for toxoplasmosis was established, which was mainly based on the results of detecting anti-*T. gondii*-specific antibodies in the cat serum sample.

Recombinant antigenic proteins may be alternative sources instead of tachyzoite antigens, which are easily obtainable for the serodiagnosis of toxoplasmosis [23]. The proteins that are commonly selected as serodiagnosis antigens are surface antigens (SAG), dense granule proteins (GRA), microneme proteins (MIC), cyst matrix antigens (MAG), and rhoptry antigens (ROP) [24]. Some of these antigens are secreted from three distinct parasite organelles called micronemes, rhoptries and dense granules, and they are crucial to *T. gondii* invasion into mammalian cells [25]. Secretory protein of GRA is an essential component of the parasitophorous vacuole (PV) and PV membrane surrounding tachyzoites. This protein has been used for serologic tests in cats, and stable ELISA detection methods based on this antigen have been established and applied to clinical samples [26–28]. AMA1, which belongs to MICs, is an important component of the moving junction (MJ) [29]. This antigen plays a key role during parasite entry into host cells [30–32]. AMA1 is engaged and secreted in almost every stage of the parasite [33], with high expression, easy purification and low economic cost. Therefore, this antigen is an ideal target for diagnosis. In this study, the results demonstrate that rAMA1 can be successfully used for the serodiagnosis of *T. gondii* infection in cats and has good diagnostic value.

The serological diagnosis depends not only on the selected detection antigen but also on the sensitivity of the detection method [19]. During the chessboard titration process, the OD450 of the negative serum had a greater effect on the establishment of the entire rAMA1-iELISA, as shown in Fig. 2B, when the P/N value was maximum, the negative serum dilution multiple was 1:100; however, the absorbance value at OD450 was greater than 0.2, which was higher than the cut-off value of some ELISA methods [27, 34]. Thus, the effect of combining the negative serum with rAMA1 and rabbit anti-cat IgG H&L/HRP was repeatedly verified to define that the negative value should be less than 0.1. The determination of the P/N value should be above 2.1 according to existing reports [35]. Furthermore, the overall performance based on ROC analysis showed that rAMA1-iELISA had an appropriate sensitivity (91.7%) and specificity (93.6%) at the optimal cut-off value of 0.112 with an AUC of 0.956, which suggested a substantial agreement with commercial ELISA, with a higher overall check accuracy than GRA7-based iELISA. As shown in Table 1, for 247 serum samples, there were differences among rAMA1-iELISA, GRA7-based iELISA and commercial ELISA kit. The positive rate that rAMA1-iELISA was higher than GRA7-based iELISA and commercial ELISA kit. The rAMA1-iELISA may contain false positives. Different experimental environments and detection targets may be the reason for the difference [36, 37]. The cut-off value that was calculated with the 45 seronegative cats was 0.144, which estimates differed from the optimal cut-off value of 0.112 above. However, a lower cut-off value may result in a higher false positive rate.

The positive rate of toxoplasmosis antibodies was 27.3% in stray cats and 5.5% in domestic cats by the rAMA1-iELISA test in Jiaxing, China. According to several surveys, the rate of toxoplasmosis infection in stray cats has been much higher than that in domestic cats in recent years; for example, the positive rate of *T. gondii* in domestic cats was 3.8% in Xiamen [38], 8.5% in Jiaxing [39], 10% in Yenning [40], and 35% globally [41], and the positive rate of *T. gondii* in stray cats was 24.4% in Jiaxing [39], 40% in Yenning [40] and 59% globally [41]. The reason for the higher seroprevalence rate of stray cats seems to be closely related to their ecological habits, such as poor living conditions and accidental eating of food containing *T. gondii*, and the parasite is usually more prevalent in moist, warm and low-altitude regions [42, 43]. Jiaxing is low and flat and located in the Yangtze River Delta plain centre, near Hangzhou Bay, Qiandao Lake and Taihu Lake [44]. Thus, water contaminated by oocysts excreted from *T. gondii*-infected cats may lead to rapid transmission on a large scale. In addition, the fact that domestic cats have a positive rate of 5.5% suggests that pet cats are at risk of *T. gondii* infection. Thus, it is of great importance to establish a reliable diagnostic method to reduce the spread of toxoplasmosis between definitive hosts and other hosts in cities. rAMA1-iELISA, a convenient and quick detection method, may be an effective means of detecting *T. gondii* infection in cats in the environment.

## Conclusion

rAMA1-iELISA method based on *T. gondii* rAMA1 was determined to have reproducibility, no cross-reactions, a low detection limit, and simple operation. Our rAMA1-iELISA method also showed that the high sensitivity, specificity and agreement with the reference technique may be found for the diagnosis of feline toxoplasmosis.

## Materials and methods

### Cat serum samples

Five domestic cats (*Felis catus*) of the Chinese Lihua breed of 6 months to 1-year old were brought from a local breeder. All cats were tested serologically by using a commercial ELISA kit (HT8012, Haitai, China) and found free of *T. gondii*. All cats were fed pasteurized milk and cooked food during the study period. A total of 1 × 10^4^ T*. gondii* RH strain tachyzoites suspended in sterile phosphate buffered saline (PBS) (BL550A, Biosharp, China) were injected intraperitoneally into three cats, and the remaining two cats were injected with sterile PBS as previously described [34]. Blood samples were collected from forelimb veins every other day after injections, and the *T. gondii*-negative and *T. gondii*-positive sera were separated and identified by using a commercial ELISA kit. Five domestic cats were anesthetized by intraperitoneal injections with a concentration of 3% pentobarbital sodium at 40 mg per 1 kg body weight then euthanized by inhaling overdose of CO_2_. Animal carcasses were autoclaved (121 ℃, 30 min) and then stored in freezers for disposal by Environmental Protection Departments. The above negative and positive cat serum samples of *T. gondii* were kindly provided by Yangzhou University.

Positive cat serum samples of FRtV, FCV and FPV were obtained from Peihao Animal Hospital (Hangzhou, China), and positive serum of *S. mansoni*, *C. sinensis* and *P. kellicotti* were kept in our lab [45, 46]. A total of 247 cat serum samples were obtained from the Shier der Animal Hospital (Jiaxing, China), of which 11 were collected from stray cats. Serum samples were kept at -80 °C until use.

### Construction of recombinant plasmid pET-32a ( +)-AMA1

Total RNA was extracted from 1 × 10^7^ tachyzoites (RH strain maintained in Vero cells) of *T. gondii* using the TRIzol Universal Reagent Kit (DP424, TIANGEN, China) and then reverse transcribed to cDNA by the First Strand cDNA Synthesis Kit (FXK-101, TOYOBO, Japan) according to the manufacturer’s instructions. Based on the gene sequence of AMA1 of *T. gondii* (GenBank: XM_002364813.1), specific PCR primers were designed to amplify the gene products of 1512 bp as follows: AMA1-F: 5’-CCGGAATTCACGTCGGGGAATCCCTTTCAG-3’; AMA1-R: 5’-CCGCTCGAGCTAGTAATCCCCCTCGACCAT-3’ by Tingke Biotechnology Company, China. The underlined letters of the primers represent the *EcoR* I and *Xho* I restriction sites. Each 50 µl amplification reaction contained 1 µl template cDNA, 25 µl 2 × PCR buffer for KOD FX, 10 µl 2 mM dNTPs, 1.5 µl from each primer (10 pmol/µl), 10 µl PCR grade water and 1 µl KOD FX (1.0 U/µl) (KFX-101, TOYOBO, Japan). PCR was conducted using the following conditions: 2 min at 94 °C for Pe-denaturation, 35 cycles of 10 s at 98 °C for denaturation, 30 s at 63 °C for annealing, 1.5 min at 68 °C for extension, and a final extension step at 68 °C for 5 min. The PCR product was cloned into the pET-32a ( +) plasmid through the *EcoR* I*/Xho* I digestion sites to generate the recombinant plasmid pET-32a ( +)-AMA1. The amplification products of PCR and enzyme digestion products of pET-32a ( +)-AMA1 were resolved on a 1% (W/V) agarose gel and stained with ethidium bromide 6 × DNA loading buffer (D0071, Beyotime, China).

### Expression and purification of rAMA1

pET-32a ( +)-AMA1 was transformed into competent *E. coli* BL21 (DE3) cells, which were cultured overnight on lysogeny broth (LB) agar plates containing 100 μg/ml ampicillin at 37 ℃. The right single colony was picked into LB medium for subculture, and then the expression of protein was induced for 0, 2, 4, and 6 h by adding 1.0 mM isopropyl β-D-1-thiogalactopyranoside (IPTG) at 37 ℃.

rAMA1 was purified according to the standard methodology. Briefly, cells were harvested by centrifugation at 8000 rpm/min for 10 min. After resuspension in 10 mM PBS, bacterial cells were disrupted by using the ultrasonicator (JY92-II, SCIZNTZ, China) with a 12 mm ultrasonic amplitude transformer with a power of 200 W/cm^2^ and a pulse of 5 s, followed by ultrasonication at low temperature 60 times. Inclusion bodies containing rAMA1 were obtained by centrifugation at 8000 rpm/min for 10 min at 4 °C. Then, 8.0 mM urea (BS124, Biosharp, China) was added to facilitate extraction for cell lysis. The bacterial solution was subjected to a Ni‐NTA Sefinose ™ Resin column after filtration with a 0.45 μm microprous membrane (BS-PES-45, Biosharp, China) for purification. Finally, the His-tagged proteins were eluted 2 ml with imidazole at different concentrations (50 mM, 100 mM, 200 mM, 400 mM, 500 mM) for further confirmation.

The expression and purity of rAMA1 were analysed through sodium dodecyl sulfate–poly-acrylamide gel electrophoresis (SDS‒PAGE) with a 12% gel. Western blotting was then carried out to evaluate the immunogenicity of rAMA1 by incubation with HRP-conjugated anti-His-Tag mouse monoclonal antibody (CW0285 M, CWBIO, China). Finally, the purified rAMA1 was quantified by a Bradford Protein Assay Kit (P0006, Beyotime Biotechnology, China).

### Optimization of the iELISA method

The iELISA method was established and optimized for detecting *T. gondii* antibodies in cats. Positive and negative controls (cat serum) were previously analysed by the commercial ELISA kit in each run below. Briefly, 96-well flat-bottom microtiter plates (BKM110308003, BKMAM, China) were coated overnight at 4 °C with purified rAMA1 diluted in 50 mM carbonate buffer (pH 9.6). The 96-well plates were blocked at 37 °C for 1 h with 1.0% BSA dissolved in PBST. After washing five times with PBST, cat serum and rabbit anti-cat IgG H&L/HRP diluted with dilution buffer (5.0% Blotting Grade dissolved in PBST) (Blotting Grade, P0216, Beyotime Biotechnology, China) were added into each well as first and second antibodies, respectively. After incubation and washing as described above, the color was developed at 37 °C for 10 min in a dark environment by the addition of 50 µl per well of TMB substrate solution for iELISA. Finally, the optical density value at 450 nm (OD450) was measured with a Bio-Tek synergy 2 microplate reader (Bio-Tek, USA) once stopped by adding 50 µl per well of 2 M H_2_SO_4_. Each serum sample had two parallel wells, and each well was read twice.

To optimize the dilution ratio of rAMA1 and cat serum, a chessboard titration, as described by Alice V. Lin [47], was performed with different qualities of rAMA1 (0.5, 1.0, 2.0, 3.0, 4.0, 5.0 μg) and different dilutions of *T. gondii*-positive and -negative cat serum samples (1:100, 1:200, 1:400, 1:800, 1:1600, 1:3200, 1:6400). The optimal rAMA1 and serum dilution were determined by the maximum ratio of positive/negative serum (P/N) value, based on the principle that the negative value was less than 0.1 and the P/N value should be above 2.1. Similarly, the incubation time (30, 60, 90 and 120 min), dilution buffer (5% Blotting Grade, 0.5%, 1.0%, and 2.0% BSA dissolved in PBST), dilution ratio of rabbit anti-cat IgG H&L/HRP (1:1000, 1:2000, 1:4000, 1:6000, 1:8000, 1:10,000 and 1:12,000), and reaction time of TMB substrate solution (5, 10, 15, 20, 25 and 30 min) were optimized by chessboard titrations as described above. In addition, the cut-off value of rAMA1-iELISA was determined by referring to the mean value ($\overline{x }$) and standard difference (SD) of OD450 of 45 negative cat serum of *T. gondii* (commercial ELISA kit verified) under optimal conditions (cut-off = $\overline{x }$  + 3SD).

### Cross-reaction, detection limit and intra-and inter-assay precision of rAMA1-iELISA

The cross-reactions of rAMA1-iELISA were assessed by using every one sample of the positive serum of common cat parasites or viruses with a dilution of 1:400 including *S. mansoni*, *C. sinensis*, *P. kellicotti*, FRtV, FCV, and FPV, and the positive cat serum of *T. gondii* was included as a control. The lowest detection limit of rAMA1-iELISA was determined by measuring serially diluted positive cat serum. To estimate the inter-and intra-assay precision, the CVs of repeated tests within batches and between batches respectively were all calculated for 5 times by using the positive and negative cat serum samples.

### Western blot analysis

rAMA1 was separated by SDS-PAGE, and the gel was transferred onto an Immobilon-PSQ PVDF membrane. The PVDF membrane was blocked overnight by 5% Blotting Grade dissolved in TBST (20 mM Tris–HCl, 150 mM NaCl, 0.05% (V/V) Tween) at 4 °C, followed by overnight incubation of *T. gondii* positive and negative controls (cat serum), cat serum samples with a dilution at 1:200 at 4 °C. After washing five times, the PVDF membrane was incubated with rabbit anti-cat IgG H&L/HRP diluted 1:4000 in 5% Blotting Grade dissolved in TBST for 1 h at room temperature and finally detected with a Hypersensitive ECL chemiluminescence kit (P0018, Beyotime Biotechnology, China).

### Clinical sample analysis

A total of 247 cat serum samples were tested by rAMA-iELISA, WB methods and a commercial ELISA kit (HT8012, Haitai, China) [48]. In parallel, GRA7-based ELISA was applied to detect cat serum samples exactly as described by Suwan et al. [22], including the construction of recombinant GRA7 plasmid, the expression and purification of recombinant GRA7 protein, establishment of indirect ELISA, and the detection of 247 cat serum samples. Commercial ELISA kit was the comparative test, rAMA1-iELISA and GRA7-based ELISA were the alternative test in all analyses. The results were subsequently analysed.

### Statistical analysis

All statistical analyses were conducted using IBM SPSS Statistics 25.0. The accuracy of the area under the curve (AUC), sensitivity and specificity of rAMA1-iELISA were evaluated by using an ROC curve [49]. The degree of agreement between GRA7-based iELISA and rAMA1-iELISA tests was evaluated by Cohen kappa coefficient statistics, and the kappa value (*κ*) was interpreted as follows: 0.0—0.20 indicated slight agreement; 0.21—0.40 indicated fair agreement; 0.41—0.60 indicated moderate agreement; 0.61—0.80 indicated substantial agreement; and 0.81—0.1 indicated perfect agreement [50].

### Supplementary Information

**Additional file 1: Supplement Figure 1.** The original figure of PCR amplification products and enzyme digestion products.

**Additional file 2: Supplement Figure 2.** The original figure of SDS-PAGE and Western blot (the blots were cut prior to hybridisation with antibodies during blotting) of expressed and analyzed recombinant Apical membrane antigen 1 (rAMA1) of *Toxoplasma* *gondii*. (A), (B) SDS-PAGE analysis of rAMA1; (C) Western blot analysis of rAMA1: (a) the blot was imaged by the Bio-Rad ChemiDoc XRS+, (b) the blot was in the Brightfield, (c) merge of a+b.

**Additional file 3: Supplement Figure 3.** Optimization of AMA1-iELISA. (A) Optimization of blocking buffer and incubation time. (B) Optimization of cat serum dilution buffer and incubation time. (C) Optimization of TMB substrate solution incubation time.

**Additional file 4.** Cat serum samples analysed by Western blot (the blots were cut prior to hybridisation with antibodies during blotting). 1-16: positive serums for Western blot, 17-38: negative serums for Western blot. (A) the blot was imaged by the Bio-Rad ChemiDoc XRS+. (B) the blot was in the Brightfield. (C) merge of A+B.

## Data Availability

The gene sequence of AMA1 of *T. gondii* generated and/or analyzed during the current study are available in NCBI repository, GenBank: XM_002364813.1.

## Abbreviations

*T. gondii*: *Toxoplasma gondii*
rAMA1‑iELISA: Indirect enzyme-linked immunosorbent assay method based on recombinant Apical membrane antigen 1
GRA7-based iELISA: Indirect enzyme-linked immunosorbent assay method based on recombinant dense granule proteins 7
PBST: 10 MM Phosphate Buffered Saline with 0.05% Tween 20
BL21(DE3): *Escherichia coli* Expression cells
IPTG: Isopropyl β-D-1-thiogalactopyranoside
ROC: Receiver operating characteristic curve
AUC: Accuracy of area under the curve

## References

[1] NasiruWana M, MohdMoklas MA, Watanabe M, Nordin N, ZasmyUnyah N, AlhassanAbdullahi S, Ahmad IssaAlapid A, Mustapha T, Basir R, Abd Majid R (2020). A review on the prevalence of Toxoplasma gondii in humans and animals reported in Malaysia from 2008–2018. Int J Environ Res Public Health.

[2] Montoya JG, Liesenfeld O (2004). Toxoplasmosis. Lancet.

[3] Wang ZD, Liu HH, Ma ZX, Ma HY, Li ZY, Yang ZB, Zhu XQ, Xu B, Wei F, Liu Q (2017). Toxoplasma gondii infection in immunocompromised patients: a systematic review and meta-analysis. Front Microbiol.

[4] Rojas-Pirela M, Medina L, Rojas MV, Liempi AI, Castillo C, Perez-Perez E, Guerrero-Munoz J, Araneda S, Kemmerling U (2021). Congenital transmission of apicomplexan parasites: a review. Front Microbiol.

[5] Blader IJ, Coleman BI, Chen CT, Gubbels MJ (2015). Lytic cycle of Toxoplasma gondii: 15 years later. Annu Rev Microbiol.

[6] Mammari N, Halabi MA, Yaacoub S, Chlala H, Darde ML, Courtioux B (2019). Toxoplasma gondii modulates the host cell responses: an overview of apoptosis pathways. Biomed Res Int.

[7] Karimi P, Shafaghi-Sisi S, Meamar AR, Nasiri G, Razmjou E (2022). Prevalence and molecular characterization of Toxoplasma gondii and Toxocara cati among stray and household cats and cat owners in Tehran. Iran Front Vet Sci.

[8] Liu Q, Cao L, Zhu XQ (2014). Major emerging and re-emerging zoonoses in China: a matter of global health and socioeconomic development for 1.3 billion. Int J Infect Dis.

[9] Levy JKCP (2004). Humane strategies for controlling feral cat populations. J Am Vet Med Assoc.

[10] Kalogeropoulos D, Sakkas H, Mohammed B, Vartholomatos G, Malamos K, Sreekantam S, Kanavaros P, Kalogeropoulos C (2021). Ocular toxoplasmosis: a review of the current diagnostic and therapeutic approaches. Int Ophthalmol.

[11] Robert MG, Brenier-Pinchart MP, Garnaud C, Fricker-Hidalgo H, Pelloux H (2021). Molecular diagnosis of toxoplasmosis: recent advances and a look to the future. Expert Rev Anti Infect Ther.

[12] Khan AH, Noordin R (2020). Serological and molecular rapid diagnostic tests for Toxoplasma infection in humans and animals. Eur J Clin Microbiol Infect Dis.

[13] Parker ML, Boulanger MJ (2015). An extended surface loop on Toxoplasma gondii Apical Membrane Antigen 1 (AMA1) governs ligand binding selectivity. PLoS One.

[14] Gatkowska J, Dzitko K, Ferra BT, Holec-Gasior L, Kawka M, Dziadek B (2020). The immunogenic and immunoprotective activities of recombinant chimeric T. gondii proteins containing AMA1 antigen fragments. Vaccines (Basel).

[15] Dautu G, Munyaka B, Carmen G, Zhang G, Omata Y, Xuenan X, Igarashi M (2007). Toxoplasma gondii: DNA vaccination with genes encoding antigens MIC2, M2AP, AMA1 and BAG1 and evaluation of their immunogenic potential. Exp Parasitol.

[16] Santos JM, Ferguson DJ, Blackman MJ, Soldati-Favre D (2011). Intramembrane cleavage of AMA1 triggers Toxoplasma to switch from an invasive to a replicative mode. Science.

[17] Lagal V, Dinis M, Cannella D, Bargieri D, Gonzalez V, Andenmatten N, Meissner M, Tardieux I (2015). AMA1-deficient Toxoplasma gondii parasites transiently colonize mice and trigger an innate immune response that leads to long-lasting protective immunity. Infect Immun.

[18] Roozbehani M, Falak R, Mohammadi M, Hemphill A, Razmjou E, Meamar AR, Masoori L, Khoshmirsafa M, Moradi M, Gharavi MJ (2018). Characterization of a multi-epitope peptide with selective MHC-binding capabilities encapsulated in PLGA nanoparticles as a novel vaccine candidate against Toxoplasma gondii infection. Vaccine.

[19] Ferra B, Holec-Gasior L, Gatkowska J, Dziadek B, Dzitko K (2020). Toxoplasma gondii recombinant antigen AMA1: diagnostic utility of protein fragments for the detection of IgG and IgM antibodies. Pathogens.

[20] Ding H, Gao YM, Deng Y, Lamberton PH, Lu DB (2017). A systematic review and meta-analysis of the seroprevalence of *Toxoplasma gondii* in cats in mainland China. Parasit Vectors.

[21] Dubey JP (2009). History of the discovery of the life cycle of Toxoplasma gondii. Int J Parasitol.

[22] Suwan E, Chalermwong P, Rucksaken R, Sussadee M, Kaewmongkol S, Udonsom R, Jittapalapong S, Mangkit B (2022). Development and evaluation of indirect enzyme-linked immunosorbent assay using recombinant dense granule antigen 7 protein for the detection of *Toxoplasma gondii* infection in cats in Thailand. Vet World.

[23] Holec-Gasior L (2013). Toxoplasma gondii recombinant antigens as tools for serodiagnosis of human toxoplasmosis: current status of studies. Clin Vaccine Immunol.

[24] Liyanage K, Wiethoelter A, Hufschmid J, Jabbar A (2021). Descriptive comparison of ELISAs for the detection of Toxoplasma gondii antibodies in animals: a systematic review. Pathogens.

[25] Carruthers V, Sibley L (1997). Sequential protein secretion from three distinct organelles of Toxoplasma gondii accompanies invasion of human fibroblasts. Eur J Cell Biol.

[26] Ybanez RHD, Kyan H, Nishikawa Y (2020). Detection of antibodies against Toxoplasma gondii in cats using an immunochromatographic test based on GRA7 antigen. J Vet Med Sci.

[27] Cai Y, Wang Z, Li J, Li N, Wei F, Liu Q (2015). Evaluation of an indirect ELISA using recombinant granule antigen Gra7 for serodiagnosis of Toxoplasma gondii infection in cats. J Parasitol.

[28] Can H, AksoyGokmen A, Doskaya M, ErkuntAlak S, DegirmenciDoskaya A, Karakavuk M, Koseoglu AE, Karakavuk T, Gul C, Guvendi M, Gul A, Guruz AY, Kaya S, Mercier A, Un C (2022). Development of a new serotyping ELISA for Toxoplasma gondii type II, type III and Africa 1 lineages using in silico peptide discovery methods, well categorized feline and human outbreak serum samples. BMC Infect Dis.

[29] Collins CR, Blackman MJ (2011). Apicomplexan AMA1 in host cell invasion: a model at the junction?. Cell Host Microbe.

[30] Lamarque M, Besteiro S, Papoin J, Roques M, Vulliez-Le Normand B, Morlon-Guyot J, Dubremetz JF, Fauquenoy S, Tomavo S, Faber BW, Kocken CH, Thomas AW, Boulanger MJ, Bentley GA, Lebrun M (2011). The RON2-AMA1 interaction is a critical step in moving junction-dependent invasion by apicomplexan parasites. PLoS Pathog.

[31] Guerin A, El Hajj H, Penarete-Vargas D, Besteiro S, Lebrun M (2017). RON4L1 is a new member of the moving junction complex in Toxoplasma gondii. Sci Rep.

[32] Lamarque MH, Roques M, Kong-Hap M, Tonkin ML, Rugarabamu G, Marq JB, Penarete-Vargas DM, Boulanger MJ, Soldati-Favre D, Lebrun M (2014). Plasticity and redundancy among AMA-RON pairs ensure host cell entry of Toxoplasma parasites. Nat Commun.

[33] Rezaei F, Sarvi S, Sharif M, Hejazi SH, Pagheh AS, Aghayan SA, Daryani A (2019). A systematic review of Toxoplasma gondii antigens to find the best vaccine candidates for immunization. Microb Pathog.

[34] Hosseininejad M (2012). Evaluation of an indirect ELISA using a tachyzoite surface antigen SAG1 for diagnosis of Toxoplasma gondii infection in cats. Exp Parasitol.

[35] Zhuo X, Sun H, Zhang Z, Luo J, Shan Y, Du A (2017). Development and application of an indirect enzyme-linked immunosorbent assay using recombinant Mag1 for serodiagnosis of Toxoplasma gondii in dogs. J Parasitol.

[36] Hong SH, Jeong YI, Kim JY, Cho SH, Lee WJ, Lee SE (2013). Prevalence of Toxoplasma gondii infection in household cats in Korea and risk factors. Korean J Parasitol.

[37] Carvalho FR, Silva DA, Cunha-Júnior JP, Souza MA, Oliveira TC, Béla SR, Faria GG, Lopes CS, Mineo JR (2008). Reverse enzyme-linked immunosorbent assay using monoclonal antibodies against SAG1-related sequence, SAG2A, and p97 antigens from Toxoplasma gondii to detect specific Immunoglobulin G (IgG), IgM, and IgA antibodies in human sera. Clin Vaccine Immunol.

[38] Zhao R, Cai ZH, Chen Q, Chen YF, Lian YH, Xia FL, Xu L (2022). Epidemiological investigation on Brucella, Toxoplasma gondii, influenza virus A and Bartonella in pet dogs and cats in Xiamen City. Chin Anim Health Inspect.

[39] Yu HJ, Jia Y, Zhu TL, Pan CM, Shen CC (2018). Seroepidemiological investigation of toxoplasmosis in dogs and cats in Jiaxing. Today Anim Husb Vet Med.

[40] Xia CF, Gao L, Wei B, Tian T (2018). Epidemiological Investigation of Toxoplasmosis of dogs and cats in Yining City. J Anhui Agric Sci.

[41] Montazeri M, MikaeiliGaleh T, Moosazadeh M, Sarvi S, Dodangeh S, Javidnia J, Sharif M, Daryani A (2020). The global serological prevalence of Toxoplasma gondii in felids during the last five decades (1967–2017): a systematic review and meta-analysis. Parasit Vectors.

[42] Foroutan M, Fakhri Y, Riahi SM, Ebrahimpour S, Namroodi S, Taghipour A, Spotin A, Gamble HR, Rostami A (2019). The global seroprevalence of Toxoplasma gondii in pigs: a systematic review and meta-analysis. Vet Parasitol.

[43] Meerburg BG, Kijlstra A (2009). Changing climate-changing pathogens: Toxoplasma gondii in North-Western Europe. Parasitol Res.

[44] Britannica, The Editors of Encyclopaedia. “Jiaxing”. Encyclopedia Britannica; 2020, https://www.britannica.com/place/Jiaxing. Accessed 28 Dec 2022.

[45] Zhuo X, Kong Q, Tong QB, Ding H, Zhang LS, Lou D, Ding JZ, Zheng B, Chen R, Wang TP, Lu SH (2019). DNA detection of Paragonimus westermani: diagnostic validity of a new assay based on loop-mediated isothermal amplification (LAMP) combined with a lateral flow dipstick. Acta Trop.

[46] Xue Y, Kong Q, Ding H, Xie C, Zheng B, Zhuo X, Ding J, Tong Q, Lou D, Lu S, Lv H (2021). A novel loop-mediated isothermal amplification-lateral-flow-dipstick (LAMP-LFD) device for rapid detection of Toxoplasma gondii in the blood of stray cats and dogs. Parasite.

[47] Lin AV (2015). Indirect ELISA. Methods Mol Biol.

[48] Li XT, Wang L, Ding Y, Sun WW (2022). *Toxoplasma gondii* infection in pet cats and their owners in northeastern China: an important public health concern. BMC Vet Res.

[49] Swets JA (1988). Measuring the accuracy of diagnostic systems. Science.

[50] Yue CJ, Yang WJ, Li YL, Zhang DS, Lan JC, Su XY, Li L, Liu YY, Zheng WC, Wu KJ, Fan XY, Yan X, Hou R, Liu SR (2022). Comparison of a commercial ELISA and indirect hemagglutination assay with the modified agglutination test for detection of Toxoplasma gondii antibodies in giant panda (Ailuropoda melanoleuca). Int J Parasitol Parasites Wildl.

