# Supplementary material for: Establishment and application of an iELISA detection method for measuring apical membrane antigen 1 (AMA1) antibodies of Toxoplasma gondii in cats
Source: BMC Vet Res. 2023 Nov 3;19:229. doi: 10.1186/s12917-023-03775-1 (PMC10623812; doi:10.1186/s12917-023-03775-1)
Supplement: Supplementary file 1 — Additional file 1: Supplement Figure 1. The original figure of PCR amplification products and enzyme digestion products. [file 12917_2023_3775_MOESM1_ESM.pdf]

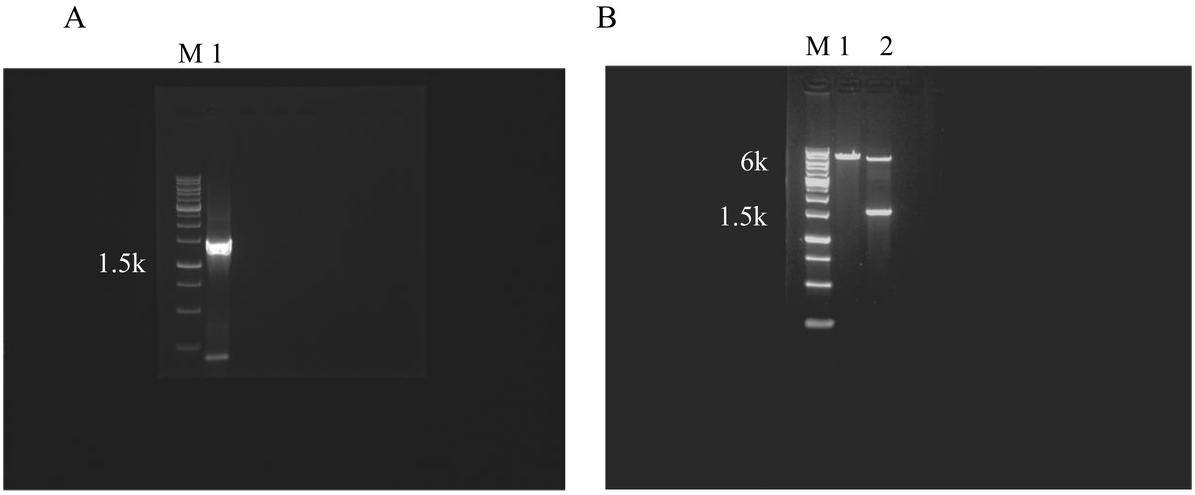


Addition file 1: Supplement Figure. 1. The original figure of PCR amplification products and enzyme digestion products.
