# Supplementary material for: Establishment and application of an iELISA detection method for measuring apical membrane antigen 1 (AMA1) antibodies of Toxoplasma gondii in cats
Source: BMC Vet Res. 2023 Nov 3;19:229. doi: 10.1186/s12917-023-03775-1 (PMC10623812; doi:10.1186/s12917-023-03775-1)
Supplement: Supplementary file 2 — Additional file 2: Supplement Figure 2. The original figure of SDS-PAGE and Western blot (the blots were cut prior to hybridisation with antibodies during blotting) of expressed and analyzed recombinant Apical membrane antigen 1 (rAMA1) of Toxoplasma gondii. (A), (B) SDS-PAGE analysis of rAMA1; (C) Western blot analysis of rAMA1: (a) the blot was imaged by the Bio-Rad ChemiDoc XRS+, (b) the blot was in the Brightfield, (c) merge of a+b. [file 12917_2023_3775_MOESM2_ESM.pdf]

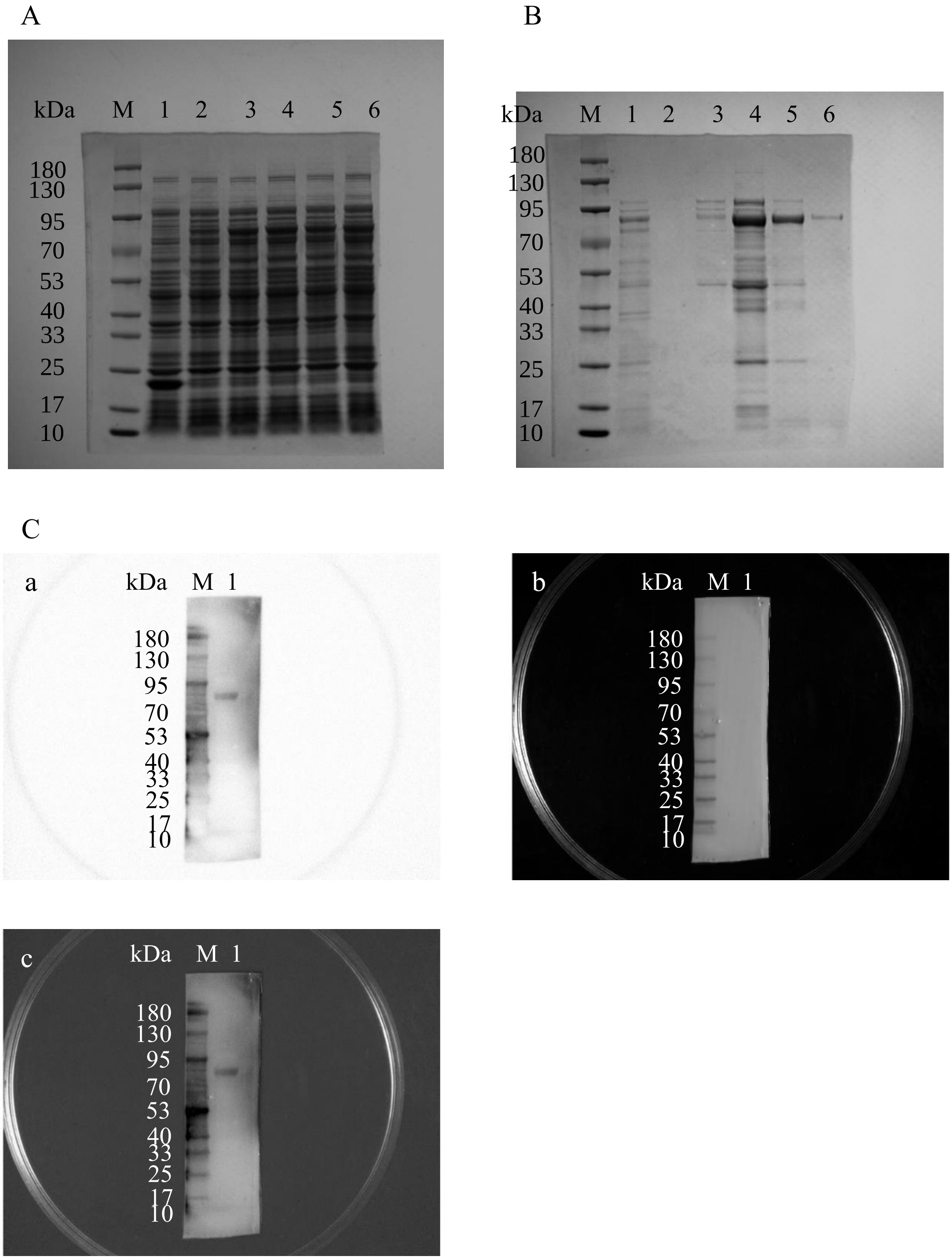

Additional file 2: Supplement Figure. 2. The original figure of SDS-PAGE and Western blot (the blots were cut prior to hybridisation with antibodies during blotting) of expressed and analyzed recombinant Apical membrane antigen 1 (rAMA1) of *Toxoplasma gondii*. (A), (B) SDS-PAGE analysis of rAMA1; (C) Western blot analysis of rAMA1: (a) the blot was imaged by the Bio-Rad ChemiDoc XRS+, (b) the blot was in the Brightfield, (c) merge of a+b.
