# Supplementary material for: Establishment and application of an iELISA detection method for measuring apical membrane antigen 1 (AMA1) antibodies of Toxoplasma gondii in cats
Source: BMC Vet Res. 2023 Nov 3;19:229. doi: 10.1186/s12917-023-03775-1 (PMC10623812; doi:10.1186/s12917-023-03775-1)
Supplement: Supplementary file 3 — Additional file 3: Supplement Figure 3. Optimization of AMA1-iELISA. (A) Optimization of blocking buffer and incubation time. (B) Optimization of cat serum dilution buffer and incubation time. (C) Optimization of TMB substrate solution incubation time. [file 12917_2023_3775_MOESM3_ESM.pdf]

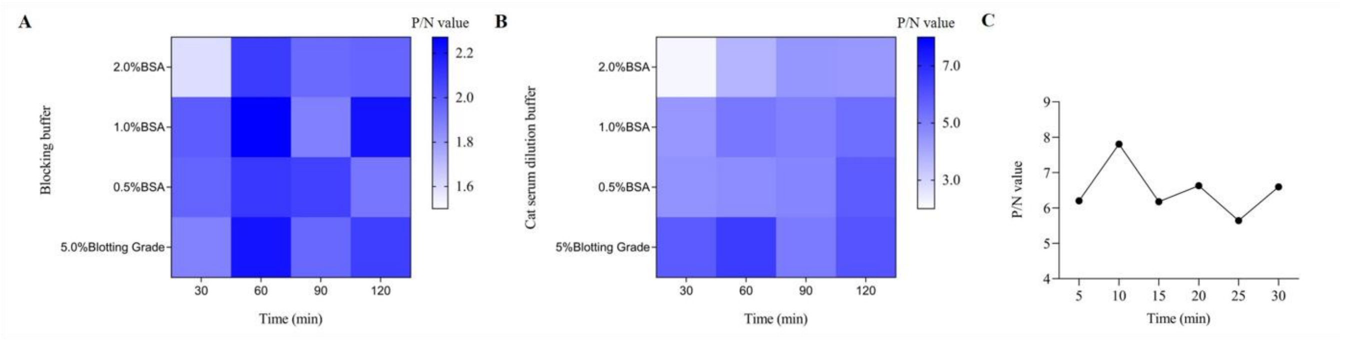

Additional file 3: Supplement Figure. 3. Optimization of AMA1-iELISA. (A) Optimization of blocking buffer and incubation time. (B) Optimization of cat serum dilution buffer and incubation time. (C) Optimization of TMB substrate solution incubation time.
