# Supplementary material for: Establishment and application of an iELISA detection method for measuring apical membrane antigen 1 (AMA1) antibodies of Toxoplasma gondii in cats
Source: BMC Vet Res. 2023 Nov 3;19:229. doi: 10.1186/s12917-023-03775-1 (PMC10623812; doi:10.1186/s12917-023-03775-1)
Supplement: Supplementary file 4 — Additional file 4. Cat serum samples analysed by Western blot (the blots were cut prior to hybridisation with antibodies during blotting). 1-16: positive serums for Western blot, 17-38: negative serums for Western blot. (A) the blot was imaged by the Bio-Rad ChemiDoc XRS+. (B) the blot was in the Brightfield. (C) merge of A+B. [file 12917_2023_3775_MOESM4_ESM.pdf]

Positive serum control-A

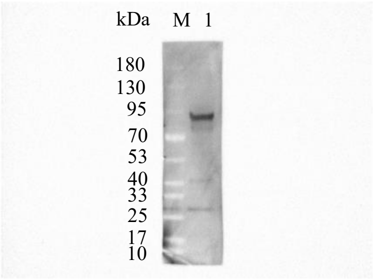

Positive serum control-B

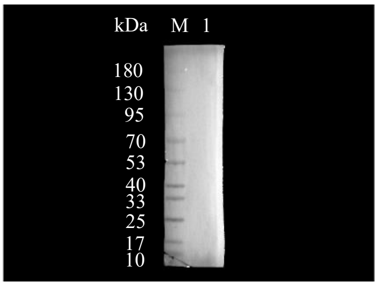

Positive serum control-C

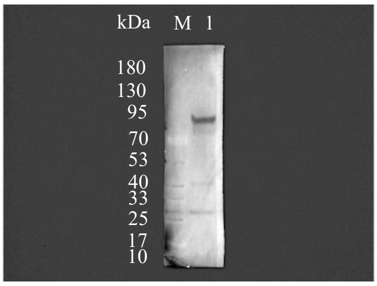

Negative serum control-A

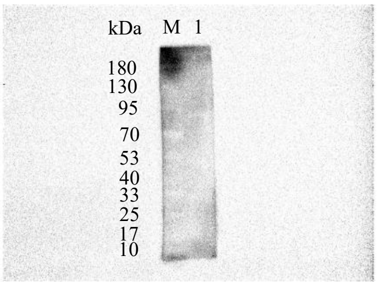

Negative serum control-B

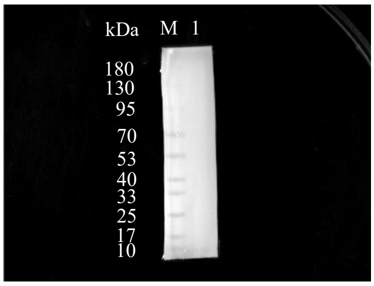

Negative serum control-C

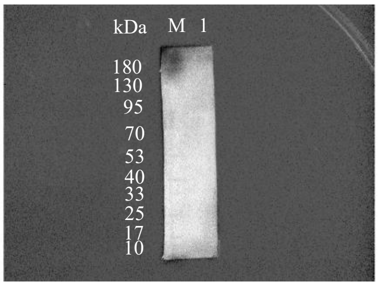

1-A

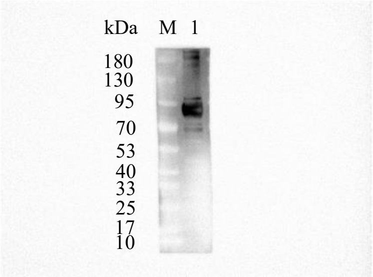

1-B

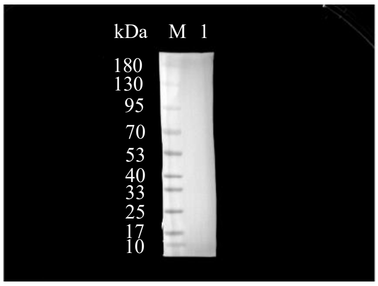

1-C

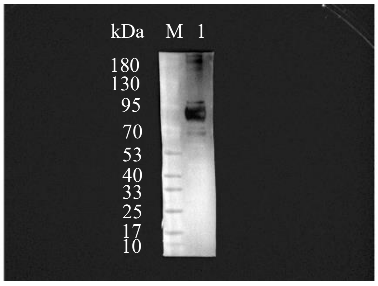

2-A

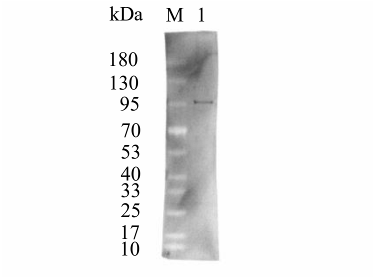

2-B

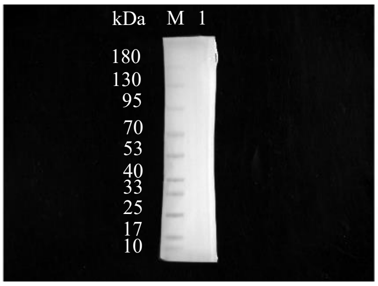

2-C

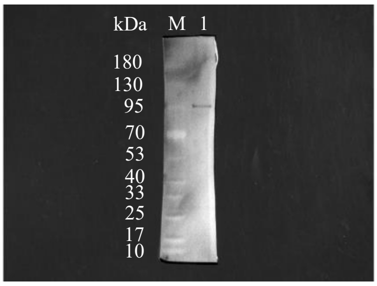

3-A

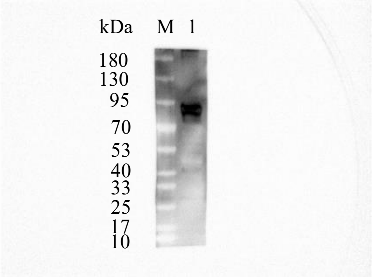

3-B

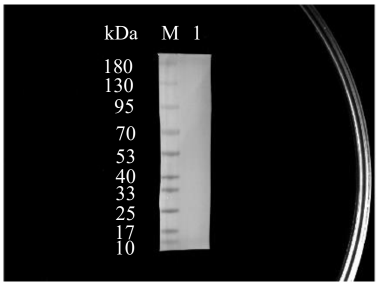

3-C

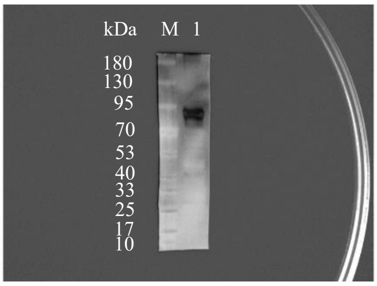

4-A

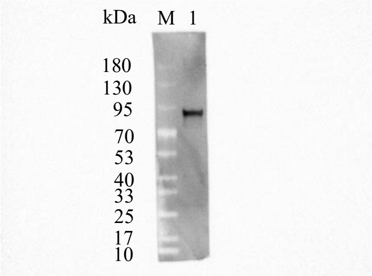

4-B

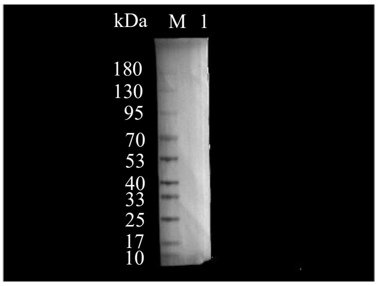

4-C

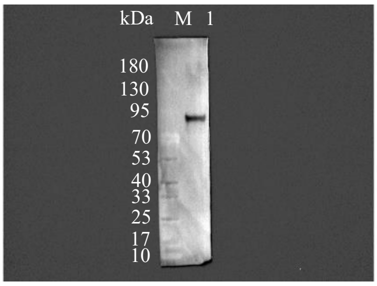

5-A

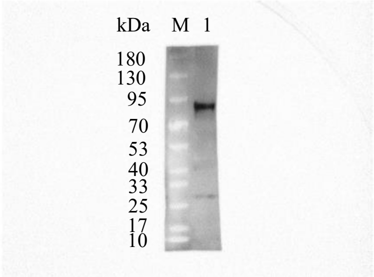

5-B

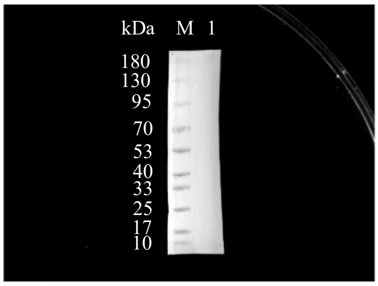

5-C

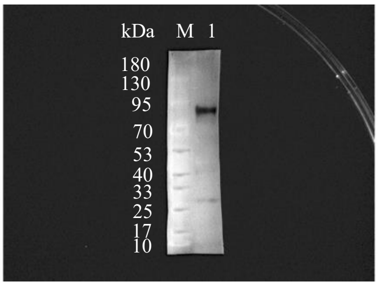

6-A

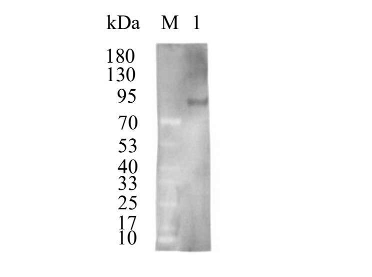

6-B

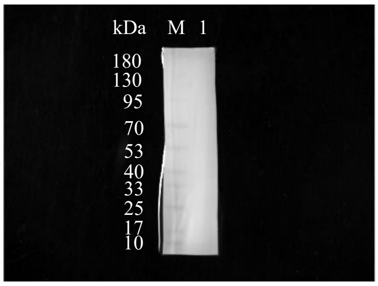

6-C

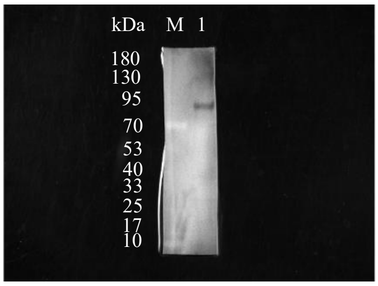

7-A

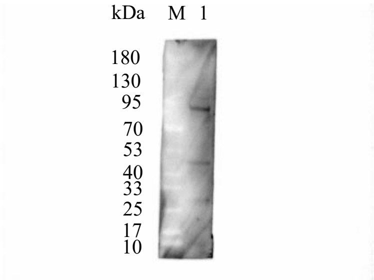

7-B

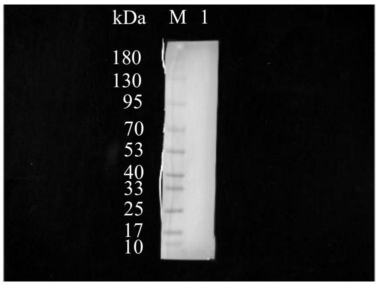

7-C

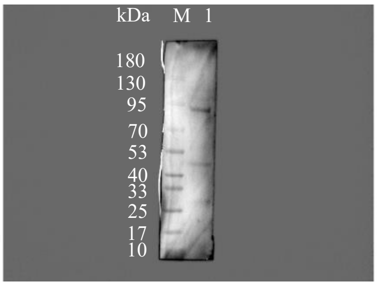

8-A

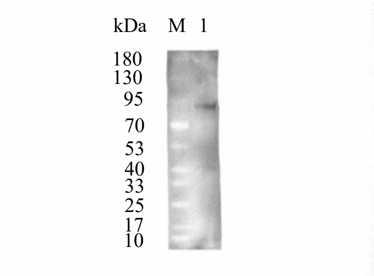

8-B

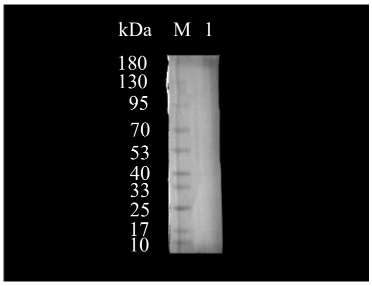

8-C

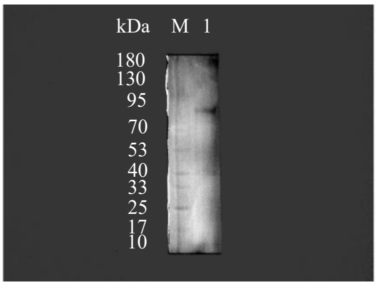

9-A

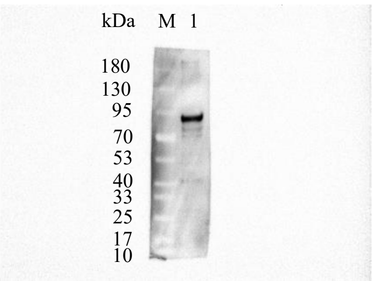

9-B

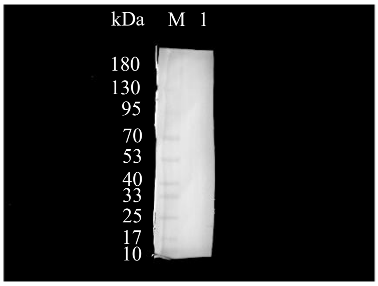

9-C

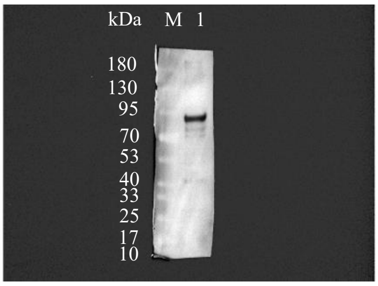

10-A

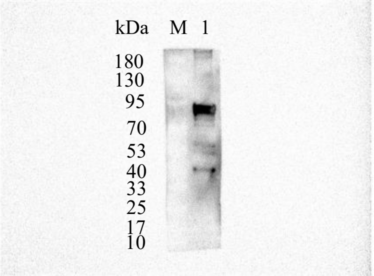

10-B

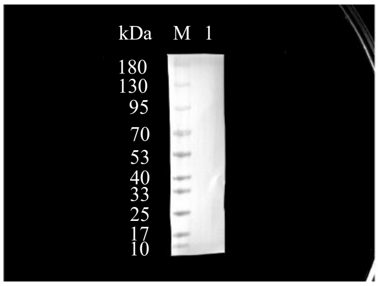

10-C

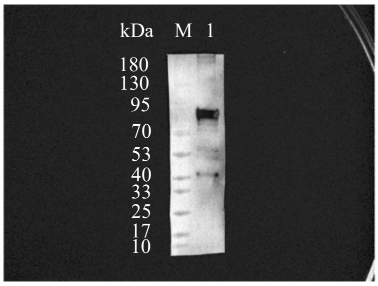

11-A

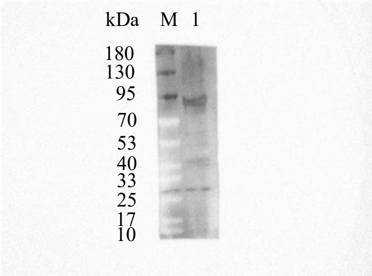

11-B

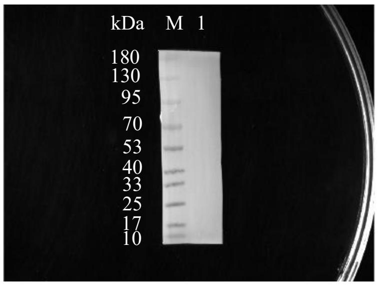

11-C

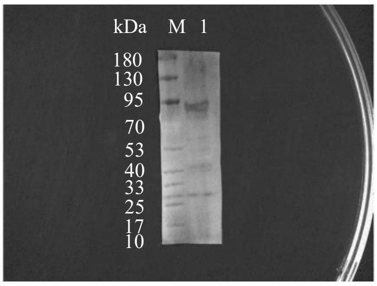

12-A

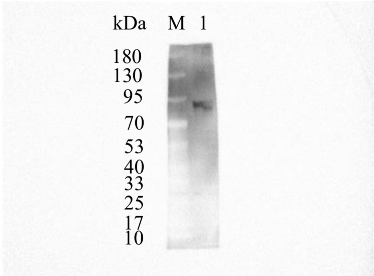

12-B

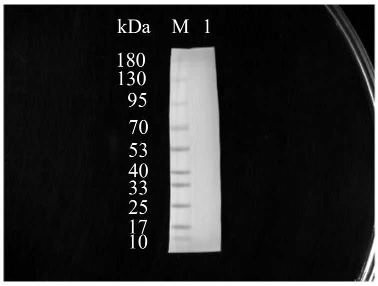

12-C

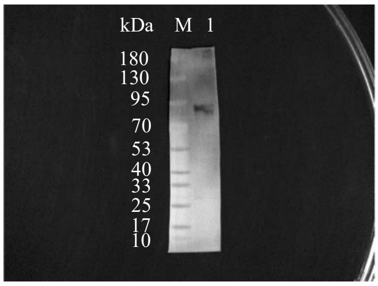

13-A

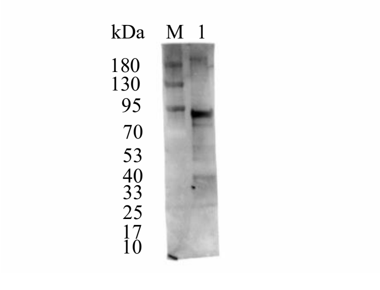

13-B

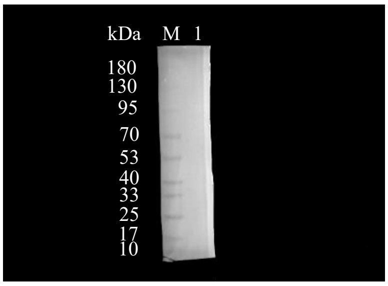

13-C

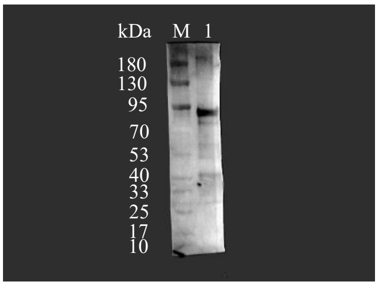

14-A

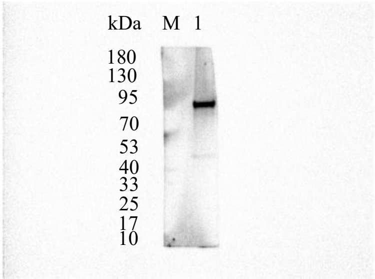

14-B

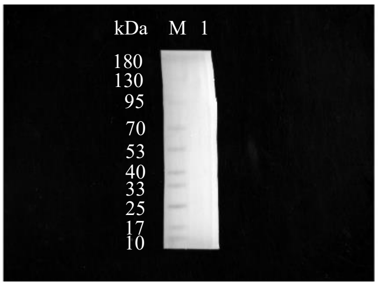

14-C

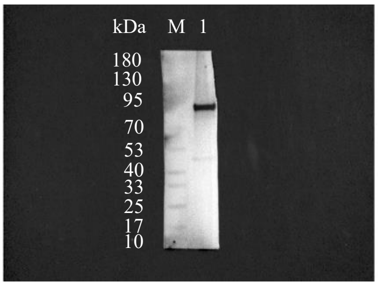

15-A

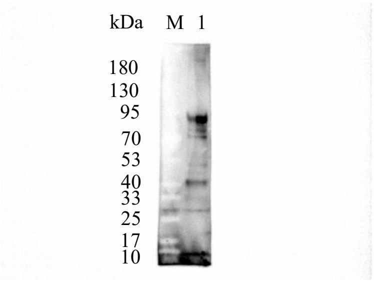

15-B

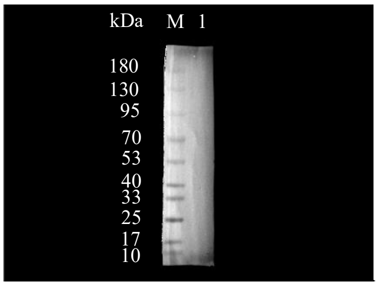

15-C

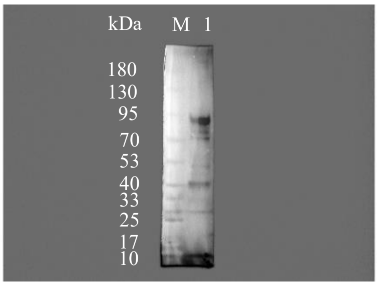

16-A

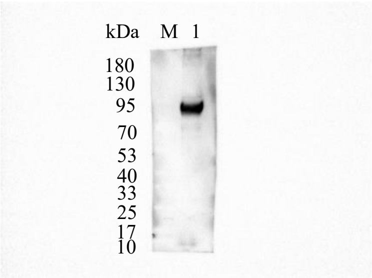

16-B

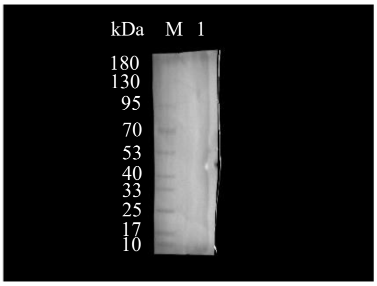

16-C

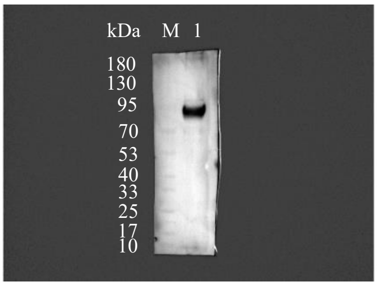

17-A

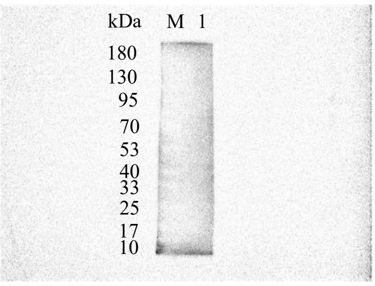

17-B

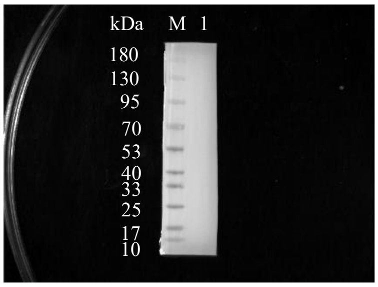

17-C

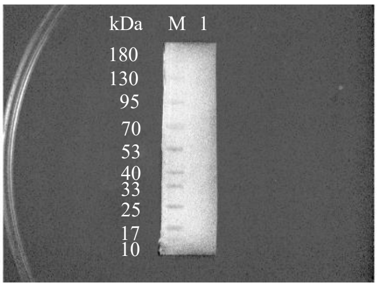

18-A

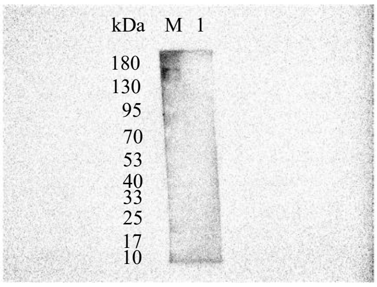

18-B

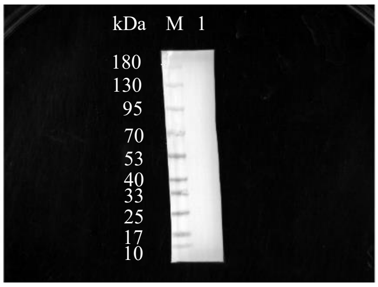

18-C

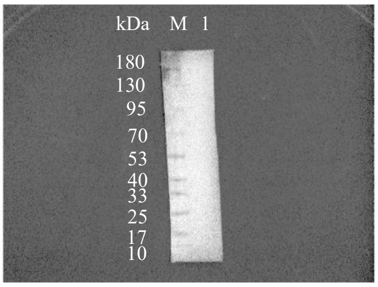

19-A

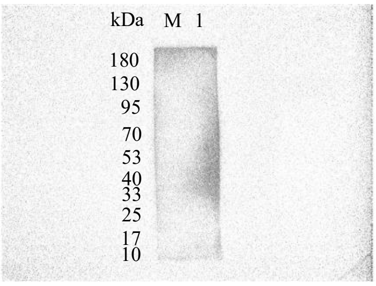

19-B

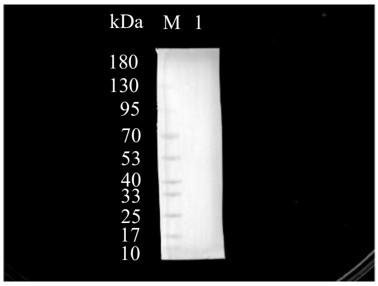

19-C

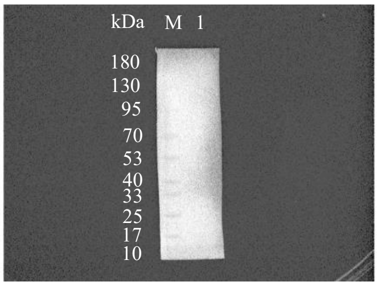

20-A

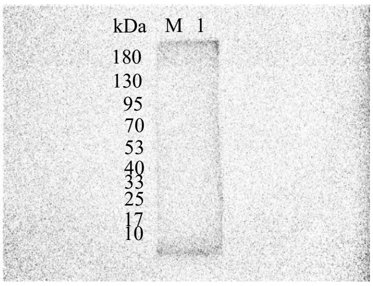

20-B

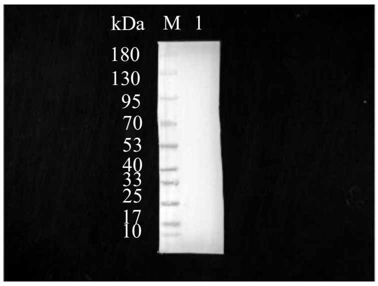

20-C

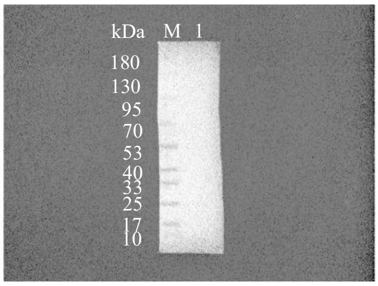

21-A

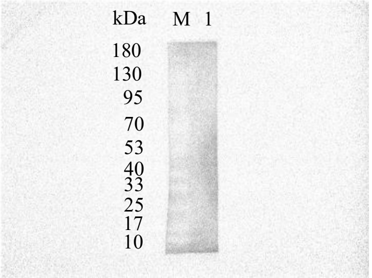

21-B

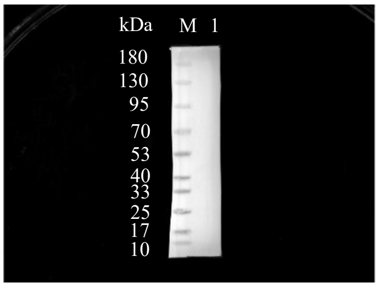

21-C

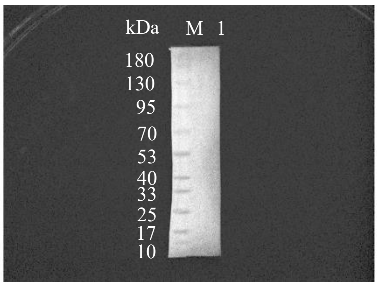

22-A

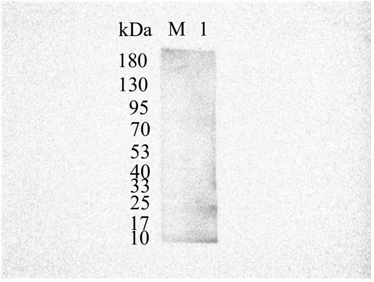

22-B

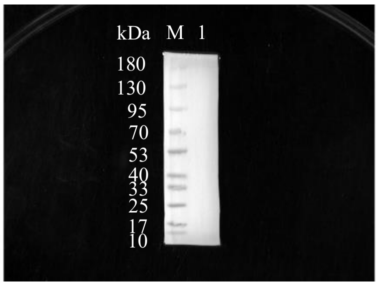

22-C

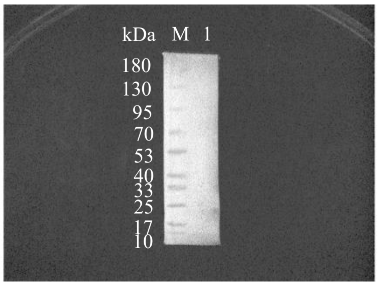

23-A

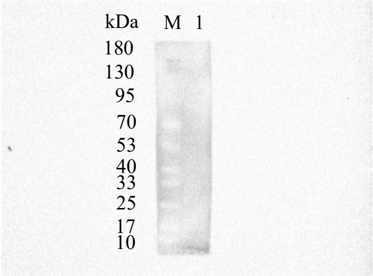

23-B

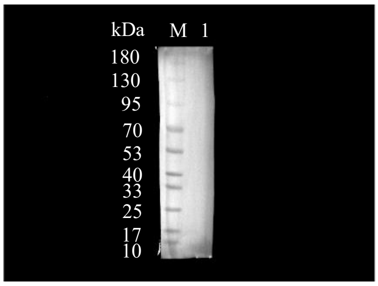

23-C

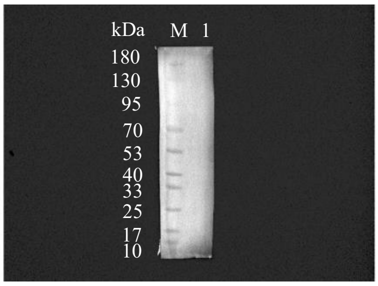

24-A

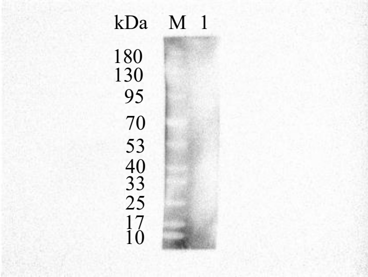

24-B

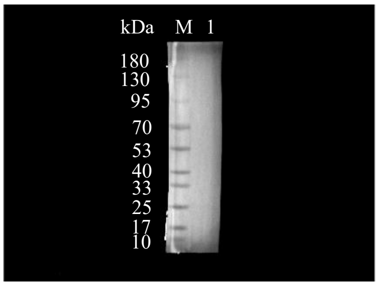

24-C

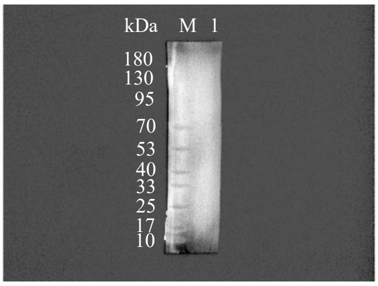

25-A

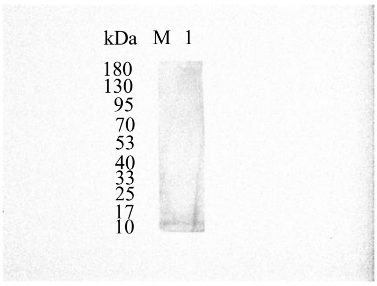

25-B

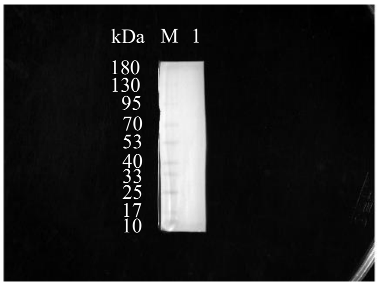

25-C

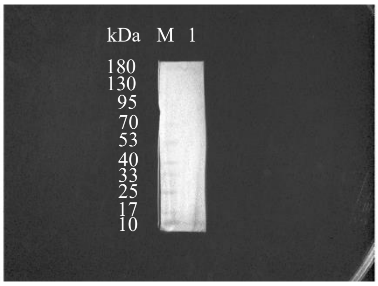

26-A

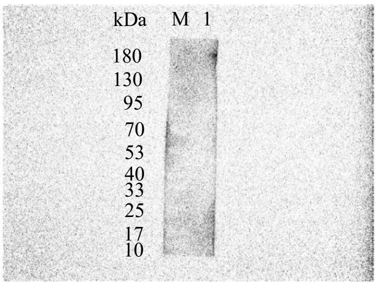

26-B

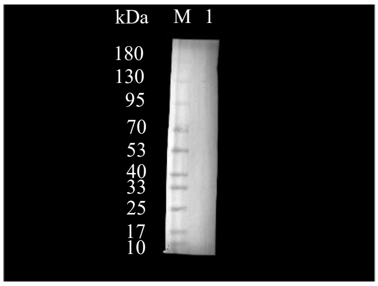

26-C

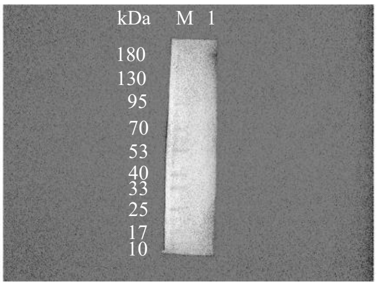

27-A

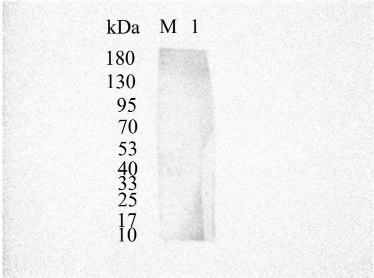

27-B

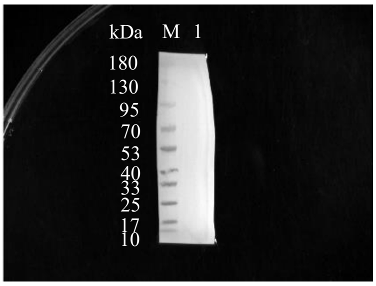

27-C

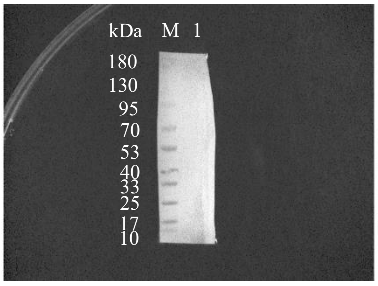

28-A

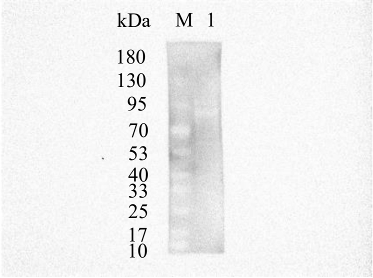

28-B

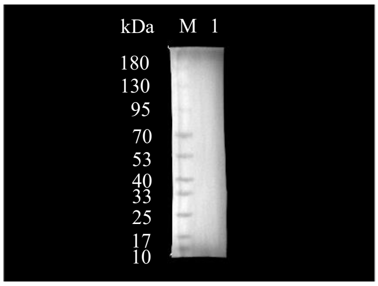

28-C

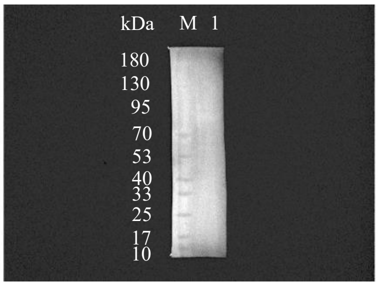

29-A

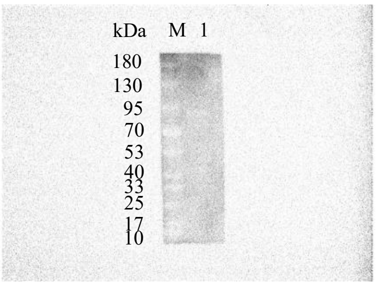

29-B

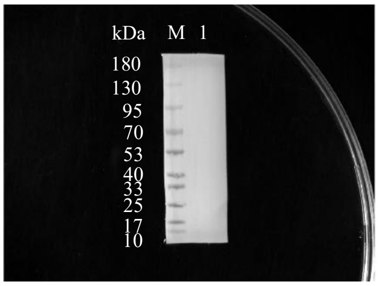

29-C

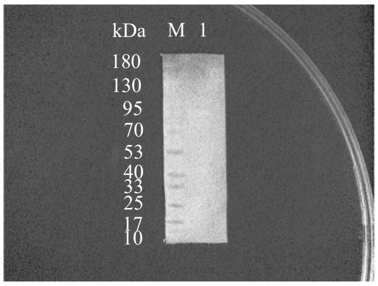

30-A

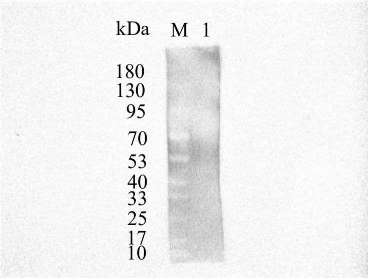

30-B

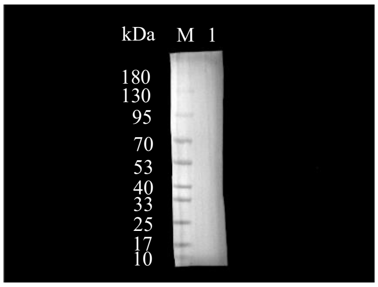

30-C

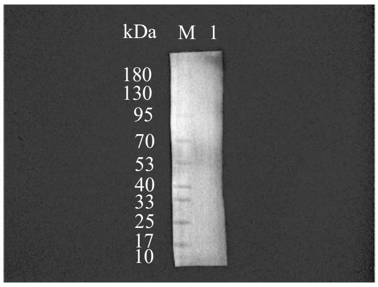

31-A

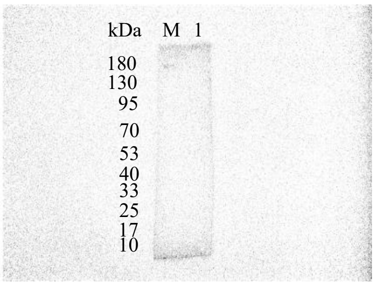

31-B

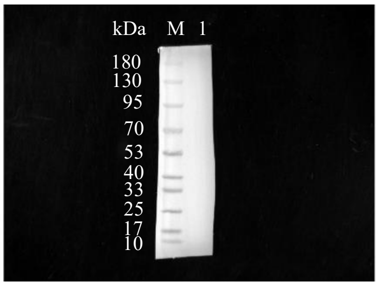

31-C

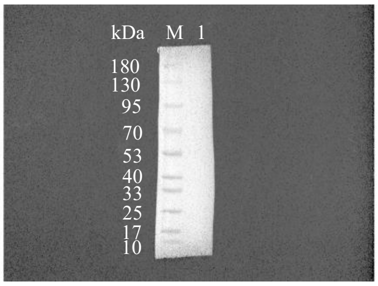

32-A

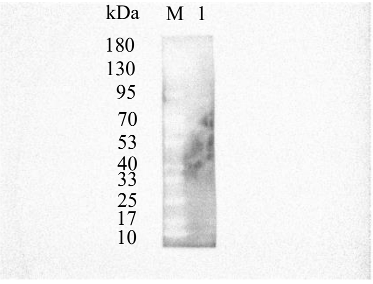

32-B

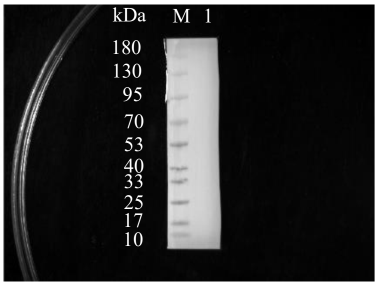

32-C

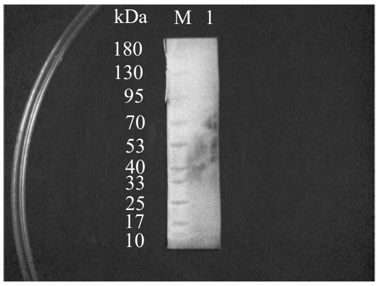

33-A

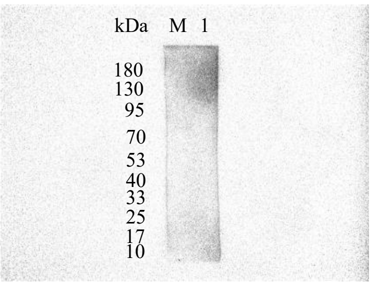

33-B

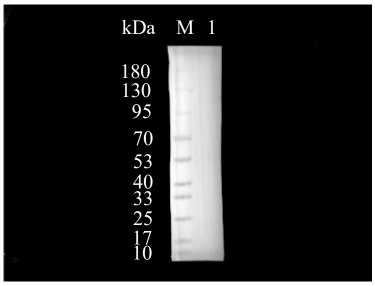

33-C

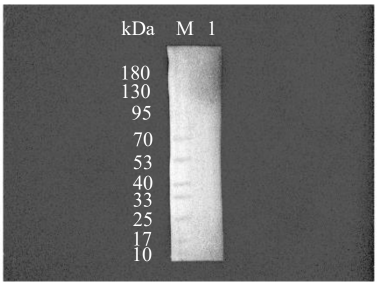

34-A

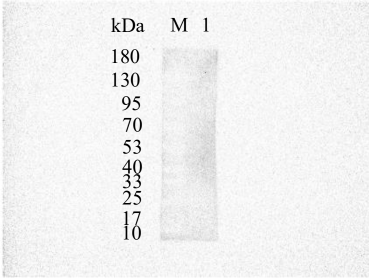

34-B

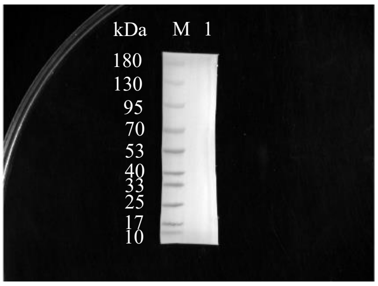

34-C

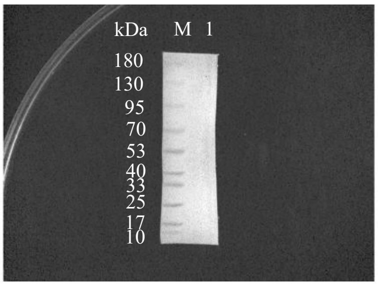

35-A

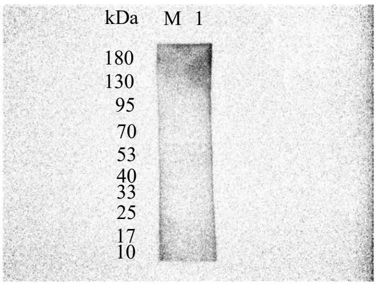

35-B

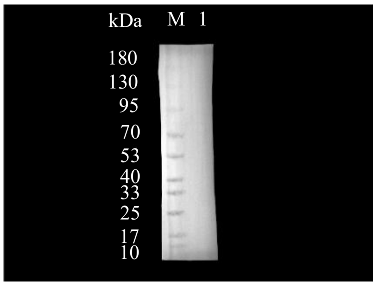

35-C

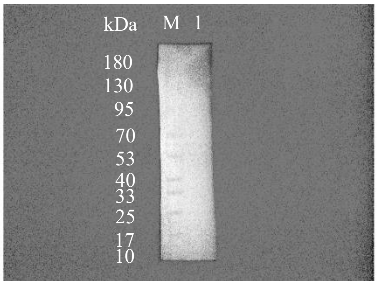

36-A

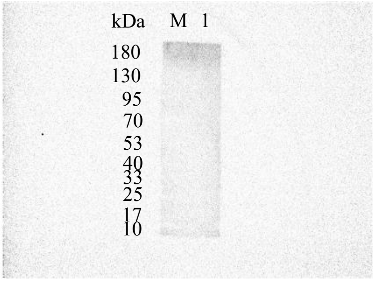

36-B

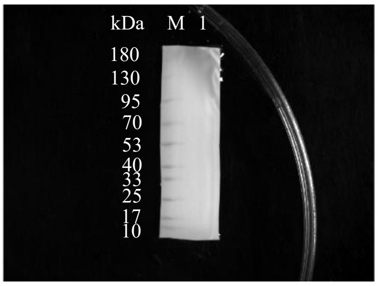

36-C

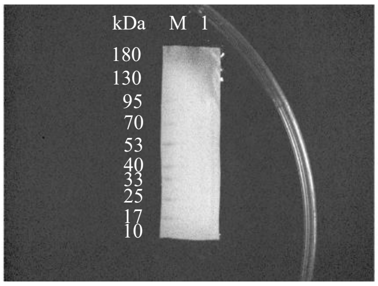

37-A

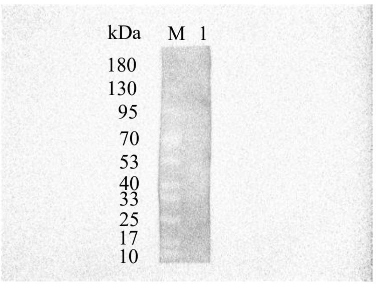

37-B

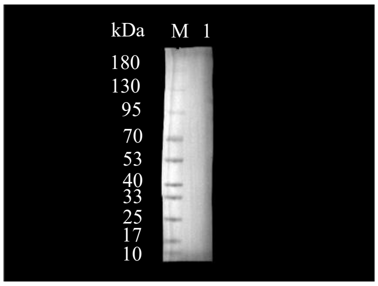

37-C

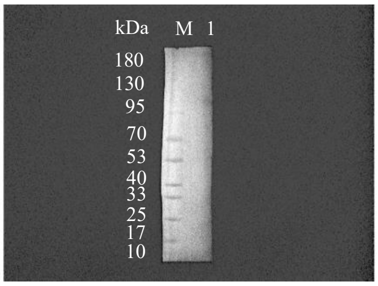

38-A

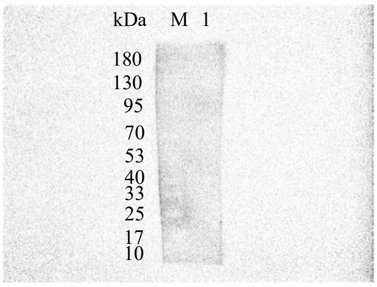

38-B

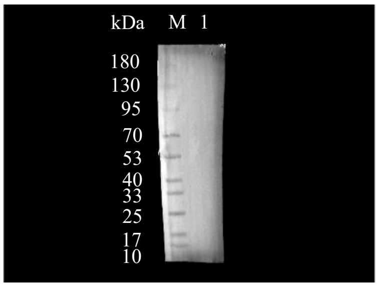

38-C

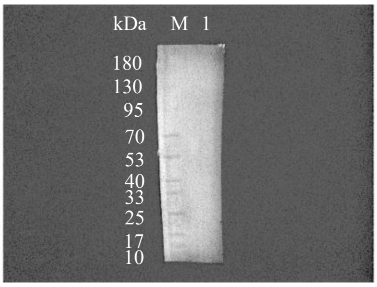

Additional file 4. Cat serum samples analysed by Western blot (the blots were cut prior to hybridisation with antibodies during blotting). 1-16: positive serums for Western blot, 17-38: negative serums for Western blot. (A) the blot was imaged by the Bio-Rad ChemiDoc XRS+. (B) the blot was in the Brightfield. (C) merge of A+B.
